# Modeling stratified dispersal in forest pests: A case study of the mountain pine beetle in Alberta

**DOI:** 10.1002/ecy.70305

**Published:** 2026-02-03

**Authors:** Evan C. Johnson, Micah Brush, Mark A. Lewis

**Affiliations:** ^1^ Mathematical and Statistical Sciences University of Alberta Edmonton Alberta Canada; ^2^ Department of Biology University of Victoria Victoria British Columbia Canada; ^3^ Department of Mathematics and Statistics University of Victoria Victoria British Columbia Canada

**Keywords:** bark beetle, black swan, *Dendroctonus ponderosae*, dispersal kernel, dispersal model, fat‐tailed distribution, forest pest, invasive species, long‐distance dispersal, range expansion

## Abstract

Forest pests pose critical threats to forest ecosystems worldwide, yet accurately predicting their spatial spread remains challenging due to complex dispersal behaviors, weather effects, and the inherent difficulty of tracking small organisms across large landscapes. These challenges have resulted in divergent estimates of typical dispersal distances across studies. Here, we use high‐quality data from helicopter and field‐crew surveys to parameterize dispersal kernels for the mountain pine beetle, a destructive pest that has recently expanded its range into Alberta, Canada. We find that fat‐tailed kernels—those which allow for a small number of long‐distance dispersal events—consistently provide the best fit to these data. Specifically, the radially symmetric Student's *t*‐distribution with parameters ρ=0.012 km and ν=1.45 stands out as parsimonious and user‐friendly; this model predicts a median dispersal distance of 60 m, with the 95th percentile of dispersers traveling nearly 5 km. The best‐fitting mathematical models have biological interpretations. The Student's *t*‐distribution, derivable as a mixture of diffusive processes with varying settling times, is consistent with observations that mountain pine beetle adults fly short distances while few travel far; early‐emerging beetles fly farther; and larger beetles from larger trees exhibit greater variance in flight distance. This phenotypic variability is mirrored in other forest pests, resulting in a stratified dispersal pattern where most individuals disperse locally while rare long‐distance “jumpers” drive range expansion. Our approach demonstrates how aerial survey data can be used to characterize dispersal patterns, as many insects create diagnostic signatures—combining foliage damage patterns and host‐tree preferences—that are visible from above. Since aerial surveys of North American forests are widely available, our methodology can be broadly used to create parsimonious dispersal models for many forest insects.

## INTRODUCTION

**FIGURE 1:**
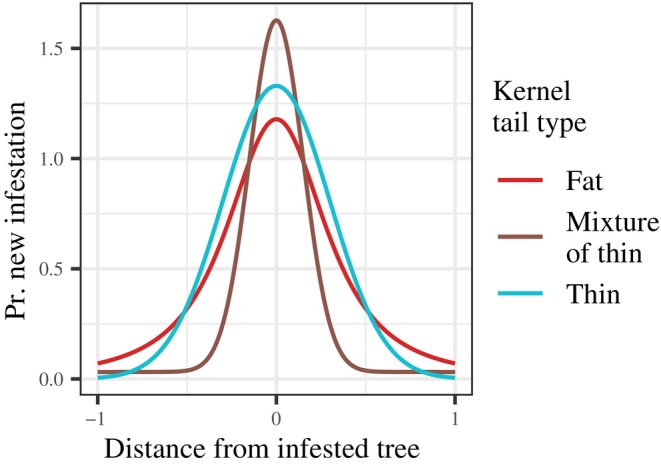
Cartoon depiction of dispersal kernels with different tail types. Fat‐tailed kernels produce a small but non‐negligible probability of long‐distance dispersal. Kernels derived from mixtures of thin‐tailed distributions (e.g., two Gaussians, respectively, with small and large SDs) can approximate fat‐tailed distributions and may allow for uniform “baseline” probability across space. Thin‐tailed kernels do not allow for long‐distance dispersal.

Dispersal is the key factor driving the spread of forest pests. Because individual movements are often unpredictable, ecologists use mathematical functions called dispersal kernels to capture variation in dispersal. The assumption of simple random motion by individuals, for instance, leads to a Gaussian “bell curve” distribution for dispersal distances (Allen, 2010; Skellam, 1951). Real dispersal data tend to look non‐Gaussian, having more long‐ and short‐distance values than found in a Gaussian distribution with comparable variance (i.e., empirical kernels are *leptokurtic*; Kot et al., 1996). The spread rate of a biological invasion is critically sensitive to the shape of the dispersal kernel (Lewis et al., 2016). In particular, long‐distance dispersal can increase the spread rate by orders of magnitude. Dispersal kernels fall into categories based on how common long‐distance movements are: thin‐tailed kernels (like the Gaussian) predict that long‐distance dispersal is extremely rare, while fat‐tailed kernels allow for more frequent long‐distance events (Figure 1). In theory, invasions driven by fat‐tailed kernels may continually increase in speed and never achieve a constant spread rate (Kot et al., 1996).

However, the dispersal of forest pests is a highly complex phenomenon that is challenging to predict (Johnson et al., 2004; Peltonen et al., 2002). Describing the flight‐based dispersal of forest pests is key to describing their spread, yet this task is confounded by biological complexity, as well as our inability to track individual dispersers. In the case of mountain pine beetle (MPB), *Dendroctonus ponderosae* Hopkins (Coleoptera: Curculionidae), dispersal is influenced by various environmental factors, including wind, temperature, and host‐tree availability (Chen & Jackson, 2017; McCambridge, 1971; Powell & Bentz, 2014). At shorter scales, their ability to aggregate and overcome host defenses is facilitated by chemical signaling (Raffa, 2001). Once a tree has been “filled up” with MPBs, additional beetles are deterred by stridulations (an auditory signal) and anti‐aggregation pheromones (Bentz et al., 1996; Safranyik & Carroll, 2006). More importantly, MPB display variable dispersal behavior: they can fly short distances through the forest to find suitable hosts (Safranyik et al., 1992), or they can disperse long distances *en masse* above the canopy, carried by convective wind currents (Hiratsuka et al., 1982a; Jackson et al., 2008). Both modes of dispersal are important at the landscape scale, but long‐distance dispersal events are relatively rare and thus difficult to predict.

Such complex dispersal processes are not unique to MPB, and can be found across a range of forest pests, including the spongy moth (*Lymantria dispar* Linnaeus), the emerald ash borer (*Agrilus planipennis* Fairmaire), and eastern tent caterpillars (*Malacosoma americanum* Fabricius) (Capinera & Barbosa, 1976; Johnson et al., 2006; Muirhead et al., 2006; Rieske & Townsend, 2005). These organisms, all of which have substantial ecological and economic impacts, are known to engage in long‐distance dispersal driven by hierarchies of complex processes (Anderson & Sturtevant, 2011). To this end, the *total dispersal kernel*, if it is possible to calculate, can be used to describe the combined influences on the overall dispersal for an individual, population, species, or community (Rogers et al., 2019).

If the total dispersal kernel is known, the mathematical theory for spreading populations could be applied, based on models coupling total dispersal and population growth dynamics, to give the spatial rate of spread for a population. However, these models generally assume no significant Allee effect (Lewis et al., 2016), defined as a decrease in per capita growth rates at low population density. Some forest pests exhibit Allee effects because they require aggregation for social feeding or mate finding (Boone et al., 2011; Raffa & Berryman, 1983; Régnière et al., 2013). Irruptive bark beetles coordinate mass attacks using aggregation pheromones, allowing groups to overwhelm individual trees' defenses. Allee effects may be negligibly small under outbreak conditions (Cooke et al., 2024), or Allee effects may be nullified through the dispersal process itself, if dispersal represents the movement of groups large enough to overcome tree defenses at their destinations. In such cases, it is actually the successful establishment of the dispersers that is of interest, and so the total dispersal kernel must be further extended to incorporate successful establishment in the form of a *total effective dispersal kernel* (TEDK). This term has been coined to describe the total dispersal kernel combined with a probability of establishment with respect to distance and direction (Schupp et al., 2010).

The question then arises whether, given the complexities of dispersal and establishment outlined above, it is even possible to determine TEDKs for forest pests. The complexities of dispersal, aggregation, and tree colonization make it difficult to use mechanistic models to reliably determine the effective dispersal (but see Garcia et al., 2022; Sturtevant et al., 2013). This key question will be the focus of our paper.

The MPB exemplifies the critical role of dispersal in forest pest invasions. The most recent outbreak of MPB is the largest bark beetle outbreak ever recorded, killing pine trees across western North America and affecting 18 million hectares in British Columbia alone (Taylor et al., 2006; Walton, 2012). Driven by fire suppression and climate change (Carroll, Régnière, et al., 2006; Creeden et al., 2014), MPB expanded its historic range in a northeasterly direction, breaching the Rocky Mountains to reach Alberta (Bleiker, 2019; Carroll & Safranyik, 2003; Nealis & Cooke, 2014). Although MPB has been currently stalled in eastern Alberta (putatively due to some combination of control efforts, host‐tree depletion, and unusually cold winters; Janice Cooke qtd. in CBC News, 2023), future outbreaks may invade pine forests stretching to the east coast of North America.

Mountain pine beetle's recent range expansion into Alberta offers a unique opportunity to study dispersal dynamics due to two key factors: data availability and the absence of endemic populations. MPB uses aggregation pheromones to coordinate mass attacks on pine trees, resulting in the rapid drying out of the trees and rust‐red foliage. This clear diagnostic feature, along with MPB's economic importance (Corbett et al., 2016), has led to extensive data collection via aerial surveys. Alberta also lacks endemic populations of MPB, with the exception of Cypress Hills (an isolated hilly region in the southeast corner of the province), thus eliminating a significant confounding factor. In other locations where endemic populations do exist, they are practically unobservable due to low densities (around 40 individuals per hectare; Carroll, Aukema, et al., 2006) and because they do not produce the diagnostic red‐topped trees; instead of mass‐attacking healthy trees, endemic beetles persist in dead or severely weakened trees with a suite of other bark beetles. Therefore, in regions where endemic populations do exist, it is often unclear whether red‐topped trees result from endemic beetles becoming able to attack healthy trees (due to climatic conditions or recent disturbances; Bleiker et al., 2014), or from epidemic beetles dispersing from elsewhere.

Dispersal analysis for forest pests takes many avenues, from empirical to theoretical, and can produce seemingly contradictory results for any given forest pest. Existing models of MPB often utilize dispersal kernels (e.g., Gaussian or Laplace distributions) that implicitly ignore long‐distance dispersal events, even though these events determine the speed of range expansion (Liu & Kot, 2019). The existing literature finds significant variation in the typical length scale of MPB dispersal (e.g., the median dispersal distance), from 10 m to 1 km to tens of km (see Table 1). Statistical models of dispersal—which use covariates like “total infestations within 3 km” rather than dispersal kernels—similarly find that typical dispersal distances vary over several orders of magnitude (e.g., Powell & Bentz, 2014; Preisler et al., 2012; Sambaraju et al., 2012). The degree of variation is so large that it plausibly represents methodological differences between studies rather than true spatial or temporal heterogeneity in beetle dispersal.

**TABLE 1:** A summary of existing literature on beetle dispersal.

Study	Scale (m)	Method	
Aukema et al. (2008)	18,000	Statistical model	
Koch et al. (2021)	17,000	Dispersal kernel	
Preisler et al. (2012)	10,000	Statistical model	
Sambaraju et al. (2012)	6000	Statistical model	
Howe et al. (2021)	5000	Statistical model	
Carroll et al. (2017)	2000	Nearest neighbor	
Simard et al. (2012)	2000	Statistical model	
Robertson et al. (2009)	1000	Modified nearest neighbor	
Strohm et al. (2013)	364	Other model	
Powell and Bentz (2014)	5–90	Other model	
Robertson et al. (2007)	30–50	Nearest neighbor	
Safranyik et al. (1992)	30	Mark–recapture	
Heavilin and Powell (2008)	10–15	Dispersal kernel	
Goodsman et al. (2016)	10	Dispersal kernel	

Data estimates from related bark beetles			Bark beetle species
Withrow et al. (2013)	1000–2500	Nearest neighbor	Douglas‐fir beetle
Turchin and Thoeny (1993)	690	Mark–recapture	Southern pine beetle
Werner and Holsten (1997)	90–300	Mark–recapture	Spruce beetle
Zumr (1992)	200	Mark–recapture	European spruce beetle
Dodds and Ross (2002)	200	Mark–recapture	Douglas‐fir beetle
Kautz et al. (2011)	100	Nearest neighbor	European spruce beetle
Zolubas and Byers (1995)	10	Mark–recapture	European spruce beetle

*Note*: We present the study, the characteristic scale of dispersal, and the methodology used. We additionally include the most direct estimates (those from mark–recapture or nearest neighbor studies) for other related bark beetles. Additional information on each of these sources and how we obtained the values in this table is available in Appendix S1: Section S3.

Mark–recapture experiments provide more direct estimates of dispersal but may underestimate typical distances. A mark–recapture experiment with MPB found typical dispersal distances of around 30 m (Safranyik et al., 1992), and other mark–recapture experiments with related beetles find similarly short distances (see Table 1). A notable shortcoming of these experiments is that they utilize pheromone traps, which may bias dispersal estimates downwards by artificially attracting beetles that would otherwise travel farther. Another approach that uses the data directly involves finding new infestations and assigning them to the closest infestation from the previous year. For brevity, we call these *nearest neighbor studies* in Table 1. Like the mark–recapture studies, nearest‐neighbor studies suffer from downward bias, and like the dispersal kernel studies, the nearest‐neighbor studies find a large range of typical dispersal distances.

Previous studies do not agree on the scale of MPB dispersal, and it can be difficult to judge individual studies without expertise in both MPB biology and modeling; therefore, there is a need for a reliable, authoritative, and easy‐to‐use dispersal model. In addition to predicting MPB infestations in the short term, describing MPB dispersal is a key stepping stone to answering long‐standing applied questions. These include (1) how spread is affected by host resistance, which is thought to vary both within and across pine species (Cudmore et al., 2010; Six et al., 2018; Srivastava & Carroll, 2023); (2) the relationship between infestation density and dispersal (Jones et al., 2019; Safranyik & Carroll, 2006); (3) the risk of MPB invasion into the high‐volume jack pine (*Pinus banksiana*) stands of western Saskatchewan; and (4) the risk of MPB invasion into the high‐volume stands of Northwest Ontario (Bleiker, 2019).

In this paper, we characterize MPB dispersal in Alberta, serving both to address specific MPB management questions and to demonstrate how aerial survey data—widely available for many regions in North America—can be used to characterize forest pest dispersal. Specifically, we fit a set of well‐established dispersal models to infestation data, and determine the TEDK with the strongest weight of evidence. We use high‐quality survey data from the Government of Alberta, which provides information on infestation locations with ±30 m positional accuracy (Government of Alberta, 2016). Because MPB were not previously present in our study areas, new outbreak locations likely represent true dispersal events rather than erupting endemic populations. We consider nine possible kernels, including thin‐ and fat‐tailed distributions. We anticipate that given the importance of long‐distance dispersal events, fat‐tailed distributions should provide the best fit to the infestation data.

## MATERIALS AND METHODS

### Background

Beetles emerge from their natal trees and attack new host trees each summer. If a tree is killed, its foliage will turn rust‐red the following summer (or early autumn), approximately 1 year after the initial attack. Knowing this diagnostic feature of year‐old infestations, the Alberta Department of Agriculture and Forestry uses helicopters to find all red‐topped trees across vast swaths of Alberta's forests. These so‐called Heli‐GPS surveys are an integral part of Alberta's management strategy for MPB; after the surveys are conducted each year (typically around September), field crews are sent to the locations of red‐topped trees. These red‐topped trees are dead and no longer contain beetles, but indicate that beetle progeny have emerged and likely attacked nearby trees. The crews then perform concentric ground surveys to find nearby green‐attack trees: trees that were recently infested and containing beetle broods. The green‐attacked trees are “controlled” (i.e., cut, and then burned or chipped) to prevent the proliferation and spread of MPB (Government of Alberta, 2016).

### Data

Our paper utilizes data from the Alberta Heli‐GPS and ground surveys, from 2005 to 2020. We restricted our main analysis to an approximately 2500 ${\mathrm{km}}^{2}$ patch of lodgepole pine (*Pinus contorta*) forest in western Alberta (Appendix S1: Figure S3). This study area was selected because it contains suitable MPB habitat (i.e., high‐biomass forest, moderate temperatures), is homogeneous compared to nearby areas (i.e., low topological complexity, contiguous forest), and was surveyed every year. For robustness, we replicated our analysis on a second 2500 ${\mathrm{km}}^{2}$ patch that is approximately 50 km east of the original study area; the results are qualitatively similar, and are thus relegated to Appendix S1: Section S1.

### Data preparation

The raw Heli‐GPS and ground survey data contain GPS coordinates and the number of trees (red‐topped or controlled trees, respectively). We rasterized these point data into 30 × 30‐m pixels. Rasterizing with a higher resolution would likely offer no benefits, seeing that location data may have positional errors up to ± 30 m.

We use $I_{t}\left(x\right)$ to denote the number of infested trees in pixel x, in year t. The number of infestations is calculated as

(1) $$I_{t}\left(x\right)=c_{t}\left(x\right)+r_{t+1}\left(x\right),$$

where $c_{t}\left(x\right)$ is the number of green‐attack trees that were located and “controlled” (i.e., burned or chipped) in the focal year, and $r_{t+1}\left(x\right)$ is the number of red‐topped trees observed in the following year. Our overarching goal is to predict the positions of next year's infestations, given this year's uncontrolled infestations, defined as $I_{t}^{*}\left(x\right)=I_{t}\left(x\right)-c_{t}\left(x\right)=r_{t+1}\left(x\right)$.

### Model

**FIGURE 2:**
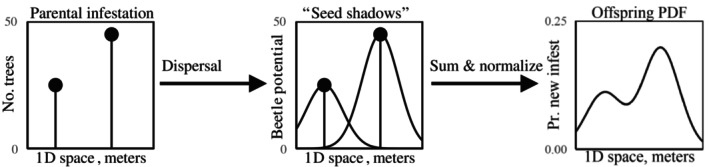
Graphical representation of our redistribution models in one dimension. A dispersal model is applied to each parental infestation, creating surfaces that represent the potential of offspring infestations stemming from parental infestations. These are called “seed shadows” in the plant ecology literature. To create a probability density function (PDF) for new infestations, we sum and normalize these seed shadows.

Conceptually, our models of MPB dispersal are simple (Figure 2). A dispersal kernel predicts the spatial distribution of infestations in the focal year (*offspring infestations*), stemming from each infestation in the prior year (*parental infestations*). All of these individualized distributions are combined to create a landscape‐scale dispersal distribution and then normalized to create a probability distribution for the offspring infestations. Unlike heuristic methods that calculate distances between offspring infestations and their closest possible parental source, the model‐based approach offers a key advantage by averaging over multiple possible origins for each offspring infestation.

We examined a variety of different sub‐models for dispersal, summarized in Table 2. The general mathematical form of dispersal kernel is $D\left(r=\text{dist}\left(x,y\right)\right)$. The function D maps the destination coordinate y and the source coordinate x to a probability density (units $1/{\text{space}}^{2}$). Here, each dispersal kernel is assumed to be radially symmetric, and thus each kernel can be parameterized as a function of the Euclidean distance between coordinates, $r=\text{dist}\left(x,y\right)$. Although we will use the terms “dispersal distance” and “dispersal kernel” for their familiarity, the jargon “redistribution distance” and “redistribution kernel” is technically more accurate, since we are not modeling beetle dispersal per se, but rather the spatial relationship between infestations.

**TABLE 2:** All of the dispersal kernels that were fit to the Alberta Heli‐GPS data, along with their tail‐type classification, functional form, and mechanistic interpretation.

Kernel name	Tail type	$D\left(r\right)\propto$	Mechanistic derivation
Pareto	Fat	${\left(r+\rho \right)}^{-\left(1+\nu \right)}$	Lévy random walk; successive dispersal steps from a mixture of distributions with disparate characteristic length scales (Benhamou, 2007)
Student's t	Fat	${\left(1+\frac{1}{v}{\left(\frac{r}{\rho }\right)}^{2}\right)}^{-\frac{v+1}{2}}$	2D Fickian diffusion with inverse‐gamma‐distributed settling times (Lewis et al., 2016, p. 167)
Bessel mixture	Mixture of Thin	$\theta \;K_{0}\left(\frac{r}{\rho_{1}}\right)+\left(1-\theta \right)K_{0}\left(\frac{r}{\rho_{2}}\right)$	2D Fickian diffusion with two different motilities and/or two different settling rates (see Bessel row below for references)
Laplace mixture	Mixture of Thin	$\theta \exp\left(-\frac{r}{\rho_{1}}\right)+\left(1-\theta \right)\exp\left(-\frac{r}{\rho_{2}}\right)$	2D Fickian diffusion with two different motilities and instantaneous settling (see Laplace row below for references)
Gaussian mixture	Mixture of Thin	$\theta \exp\left(-{\left(\frac{r}{\rho_{1}}\right)}^{2}\right)+\left(1-\theta \right)\exp\left(-{\left(\frac{r}{\rho_{2}}\right)}^{2}\right)$	2D Fickian diffusion with two different motilities and a single settling time, or a single motility constant and two different settling times (see Gaussian row below for references)
WMY	Thin	${\left(\frac{r}{\rho }\right)}^{\kappa }K_{\kappa }\left(\frac{r}{\rho }\right)$	2D Fickian diffusion with gamma‐distributed stopping times *or* 2D fractal diffusion with constant settling rate (Koch et al., 2020)
Bessel	Thin	$K_{0}\left(\frac{r}{\rho }\right)$	2D Fickian diffusion with constant settling rate (Broadbent & Kendall, 1953)
Laplace	Thin	$\exp\left(-\frac{r}{\rho }\right)$	Turbulent diffusion then instantaneous settling (Joseph & Sendner, 1958) *or* a bout of 2D Fickian diffusion followed by a bout of fractal diffusion, then instantaneous settling (Koch et al., 2020)
Gaussian	Thin	$\exp\left(-{\left(\frac{r}{\rho }\right)}^{2}\right)$	2D Fickian diffusion then instantaneous settling (Skellam, 1951)

*Note*: The Whittle–Matérn–Yasuda kernel (abbr. WMY) contains the function $K_{\kappa }$ denoting the κth order modified Bessel function of the second kind. The parameter ν is constrained to be greater than 1; otherwise, the normalizing constant of the distribution does not exist. This table was inspired by Table 1 in Koch et al. (2020).

There are three classes of dispersal kernels: thin‐tailed, fat‐tailed, and mixtures of thin‐tailed distributions. The moniker “thin‐tail” describes any distribution whose tails decay exponentially (or faster) in the limit of large r. The Gaussian distribution is a prime example of this category, exhibiting the fastest decay rate among all the distributions we examined. By contrast, fat‐tailed distributions decay slower than exponentially. The Pareto distribution is a prime example of this category, with tails so fat the variance of the distribution does not exist when the scale parameter ν<2. Mixture distributions, which are weighted averages of two thin‐tailed distributions, technically remain thin‐tailed due to their asymptotic behavior, but can simulate fat‐tailed distributions over intermediate distances by blending distributions with various distance scales. The flexible nature of mixtures is illustrated by the radially symmetric Student's *t*‐distribution, also referred to as the “2Dt dispersal kernel.” This properly fat‐tailed distribution can be derived as an infinite mixture of Gaussian distributions whose variances are prescribed to follow an inverse‐gamma distribution (Lewis et al., 2016).

The likelihood function is computed by first convolving the map of the parental infestations, $I_{t-1}^{*}\left(x\right)$, with the dispersal kernel:

(2) $$B_{t}\left(y\right)=\sum_{x}\;I_{t-1}^{*}\left(x\right)D\left(\text{dist}\left(y,x\right)\right){\left(\Delta x\right)}^{2},$$

with Δx=0.03km as the spatial resolution. The result is the *beetle potential* $B_{t}$, which represents the average number of offspring infestations arriving at each location. Note that Equation (2) is the discretization of a continuous‐space convolution; thus, $\text{dist}\left(y,x\right)$ is the distance between the centers of two 30‐m pixels, regardless of the spatial distribution of infestations within those pixels.

The beetle potential is re‐scaled to produce a likelihood surface:

(3) $$\pi_{t}\left(x\right)=\frac{B_{t}\left(x\right)}{\sum_{y}I_{t-1}^{*}\left(y\right)}.$$

While the convolution is performed for all infestations within the approximately 50 × 50 km study area, the likelihood is only calculated using the new infestations that were further than 10 km from the boundary of the study area. This buffer zone circumvents statistical edge effects, ensuring that each modeled infestation is allowed to receive virtual beetles from all directions. Each offspring infestation is treated as an independent and identically distributed random variate, and thus the log likelihood in year t is

(4) $$L_{t}=\sum_{x:I_{t}\left(x\right)>0}\;I_{t}\left(x\right)\times \log\left(\pi_{t}\left(x\right)\right).$$

The total log likelihood is simply the sum over years, from 2009 to 2019: $L=\sum_{t=1}^{11}\;L_{t}$. While the presence of MPB in Alberta dates back to 2005, the years 2009–2019 constitute the main phase of heightened MPB presence (Appendix S1: Figure S4).

All dispersal kernels were fit with maximum likelihood estimation. More specifically, multi‐parameter kernels were fit with the Nelder–Mead algorithm, whereas the single‐parameter kernels (i.e., the Laplace, Gaussian, and Bessel) were optimized using high‐resolution likelihood profiles (Appendix S1: Figure S5). All analyses were performed in R (R Core Team, 2022).

This modeling framework can be extended to accommodate multivoltine species by incorporating generation‐specific dispersal and reproductive parameters. Take, for example, the European spruce beetle (*Ips typographus* Linnaeus). In areas where this species is bivoltine, beetles emerging in the spring are more numerous and disperse farther than beetles emerging in the summer (Doležal et al., 2016; Faccoli & Stergulc, 2004). To model this system, we would convolve the first dispersal kernel with all year t−1 (uncontrolled) infestations to generate a likelihood surface representing potential first‐generation attacks in year t. Next, we would convolve this surface with a second dispersal kernel, resulting in a second likelihood surface representing second‐generation attacks. The final prediction combines both likelihood surfaces, weighted by the expected reproductive output of their respective generations—with the first surface receiving higher weight because it represents attacks by the overwintering brood, which is typically larger. This approach assumes that year t−1 infestations are equally likely to represent either generation, since we cannot distinguish between them in annual surveys.

### Model validation

The dispersal models were validated and compared using a holistic approach that combined metrics of relative model fit, absolute model fit (e.g., forecast correlation, true detection rate), and visual agreement between model predictions and salient features of the data. We used log likelihoods to compare relative model fit; given that all candidate models contain few parameters (1–3) and our large sample size (56,993 infestations), parameter penalties in information criteria like Akaike information criterion (AIC) would be negligible.

We also calculated the distance between offspring infestations and the nearest parental infestation. This nearest neighbor analysis produces a lower bound for model‐based estimates of dispersal distance, and indicates the general shape of the marginal dispersal kernel (i.e., $\int_{0}^{2\pi }D\left(r\right)\mathrm{rd}\theta =2\pi \;r\;D\left(r\right)$, where r is the distance in kilometers and θ is the angle in radians), and helps us roughly to classify observations as short‐ or long‐distance dispersal events. This classification enables us to assess models more effectively by examining the log likelihood of observations within defined categories of dispersal distance.

To explore the long‐term implications of the different dispersal kernels, we simulated the spread of MPB across Alberta. Each simulation started with the same initial conditions, the observed infestations in 2005. Then, for each successive year, the simulated infestations determine the beetle‐potential surface and corresponding probability mass function, from which the new infestations are simulated. The number of simulated infestations was equal to the actual number of observed infestations in the focal year; this methodology accounts for the fact that some years (particularly the 2008–2009 transition) exhibit variable reproductive rates, potentially because of fluctuating climatic factors. The simulation was constrained to areas where lodgepole pine is the dominant pine species and where pine biomass constitutes more than 1% of total live aboveground biomass.

## RESULTS

Across all forms of evidence, the most effective models featured fat‐tailed dispersal kernels. There were larger differences between the three categories of dispersal kernels (thin‐tailed, fat‐tailed, and mixture) than there were within each category. The differences between fat‐tailed and mixture‐based dispersal kernels are subtle, whereas models with thin‐tailed dispersal kernels consistently performed worse by all measures of quality. The total log likelihoods are highly negative (on the order of $-10^{5}$), which is a natural and expected consequence of the spatial scale of our analysis—at a resolution of 30 × 30‐m pixels, the probability that a new infestation falls within any particular pixel is very small.

**FIGURE 3:**
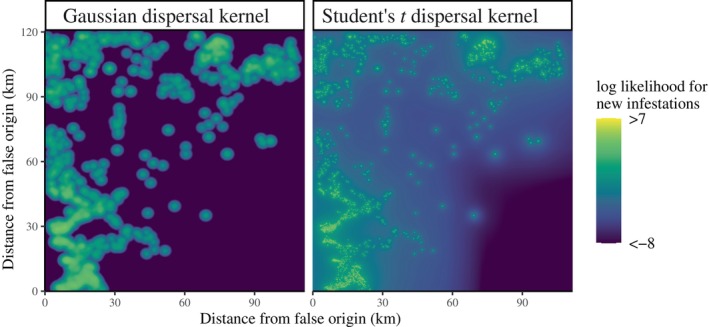
In order to account for medium‐ to long‐distance dispersal events, thin‐tailed dispersal kernels (here, the radially symmetric Gaussian) predict that offspring infestations will be several kilometers from parental infestations. The fat‐tailed dispersal kernels (here, the radially symmetric Student's t) predominantly predict short‐distance dispersal, along with a small number of long‐distance dispersal events. The maps display model predictions of beetle potential from trees that were infested in 2006. The origin has longitude and latitude coordinates −119.7269, 53.60261.

Thin‐tailed dispersal kernels, like the familiar Gaussian and Laplace kernels, consistently produced low log likelihoods, predictive correlations, and true positive rates for new infestations (Table 3). There is a large log likelihood penalty for dispersal kernels that predict a near‐zero infestation probability for a pixel in which an infestation does occur; to avoid this penalty, the thin‐tailed dispersal kernels predict that most dispersal is medium‐distance dispersal. This phenomenon is most evident in the case of the Gaussian dispersal kernel, which has the fastest‐decaying tails of any distribution considered here, and consequently predicts a median redistribution distance of 0.98 km (Table 3). This is probably wrong, since the majority of experiments and models—including our other dispersal kernels—imply that most beetles disperse much shorter distances. The general pattern, wherein thin‐tailed kernels predict a predominance of medium‐distance dispersal, is readily visualized with a 2D log‐likelihood surface (Figure 3).

**TABLE 3:** Summary statistics of model fit and redistribution distances for the redistribution models, fit to data from study area #1.

Kernel name	Log likelihood	TPR	r (log scale)	Mean dist. (km)	Median dist. (km)	75% dist. (km)	95% dist. (km)
Pareto	$-7.494\times 10^{5}$	0.009	0.327	0.925	0.076	0.296	3.772
Student's t	$-7.488\times 10^{5}$	0.015	0.330	1.037	0.061	0.259	4.529
Bessel mixture	$-7.498\times 10^{5}$	0.004	0.343	2.527	0.070	3.318	12.846
Laplace mixture	$-7.510\times 10^{5}$	0.004	0.346	3.009	0.065	4.772	13.864
Gaussian mixture	$-7.520\times 10^{5}$	0.013	0.348	4.643	1.339	8.658	15.648
WMY	$-8.049\times 10^{5}$	0.000	0.170	0.355	0.284	0.483	0.903
Bessel	$-8.050\times 10^{5}$	0.000	0.168	0.337	0.270	0.459	0.859
Laplace	$-8.183\times 10^{5}$	0.000	0.148	0.429	0.360	0.578	1.019
Gaussian	$-8.477\times 10^{5}$	0.000	0.096	0.982	0.922	1.305	1.918

*Note*: The abbreviation TPR stands for *True Positive Rate*; r is the correlation between the logarithm of the observed number of infestations and the logarithm of the expected number of infestations; and the last 4 columns refer to the distances between new infestations and their parental infestations. To compute the TPR, we identify a “positive” as one or more infestations within a 30 × 30‐m pixel, and we predict a positive when the probability of one or more infestations is greater than 1/2. This probability is computed as the complement of the Bernoulli probability of observing zero new infestations, given a number of trials equal to the total infestations observed in the focal year, and the per‐trial probability is derived from the redistribution model. The expected number of infestations (used in the calculation of r) is simply the per‐trial probability of an infestation, multiplied by the total number of infestations in the focal year.

Fat‐tailed kernels produce higher log likelihoods than mixture‐based kernels, but the difference is slight, thus suggesting that the two types of dispersal kernels must be differentiated on the basis of additional features. Both predict a majority of short‐distance dispersal and a minority of long‐distance dispersal, but the mixture models are more extreme. To see this, consider the Laplace kernel with density $\exp\left(-r/\rho \right)$, which has the convenient property that the mean distance equals 2×ρ km. The Laplace mixture model predicts that a θ=0.62 proportion of beetles engage in short‐distance dispersal with a mean dispersal distance of 42 m, while the remaining beetles disperse with a mean distance of 7.8 km (Appendix S1: Table S5).

**FIGURE 4:**
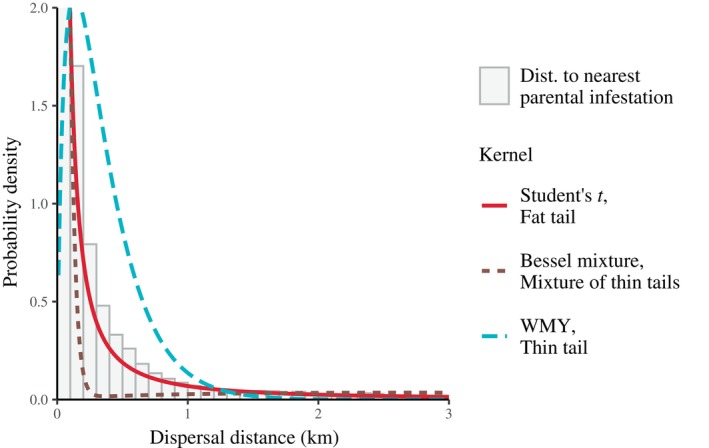
Predictions versus observations for various dispersal kernels. The histogram gives the frequency of distances between infestations and the closest “parental infestation” from the previous year. The predictions come from the marginal dispersal kernels $D_{r}\left(r\right)$, that is, $\int_{0}^{2\pi }D\left(r\right)\mathrm{rd}\theta =2\pi \;r\;D\left(r\right)$, where r is the distance in kilometers, θ is the angle in radians, and $D\left(r\right)$ is the full density (see Table 2). For each class of distributional tail—thin, mixed, and fat—we display the model with the highest log likelihood.

There are several reasons for treating fat‐tailed dispersal kernels as the default choice when modeling MPB. (1) Fat‐tailed kernels provide a superior fit to the distribution of distances between offspring infestations and the nearest parental infestations (Figure 4). (2) Mixture models are less parsimonious, with ≥3 parameters. (3) According to the distance‐stratified log likelihoods (Appendix S1: Figure S6), mixture‐based kernels are better at predicting offspring infestations that are far away from any parental infestation. However, fat‐tailed kernels are better at predicting nearby offspring infestations, which constitute the vast majority of new infestations.

## DISCUSSION

Fat‐tailed dispersal kernels, particularly the Student's *t*‐distribution, emerge as an effective base model of forest pest dispersal. The Student's *t*‐distribution has only two parameters (degrees of freedom ν and scale parameter ρ), making it simpler than mixture‐based alternatives; it can be fit using only 2 years of spatial infestation data; and most importantly, it elegantly interpolates between dispersal regimes based on a single parameter. When ν is large, the distribution approximates a Gaussian kernel that exclusively captures short‐distance dispersal. When ν approaches zero, the distribution's heavier tails allow for both short‐distance and long‐distance events (i.e., stratified dispersal). This flexibility allows the Student's *t*‐distribution to parsimoniously model the full spectrum of dispersal behaviors and phenotypic variability observed in forest pests.

**FIGURE 5:**
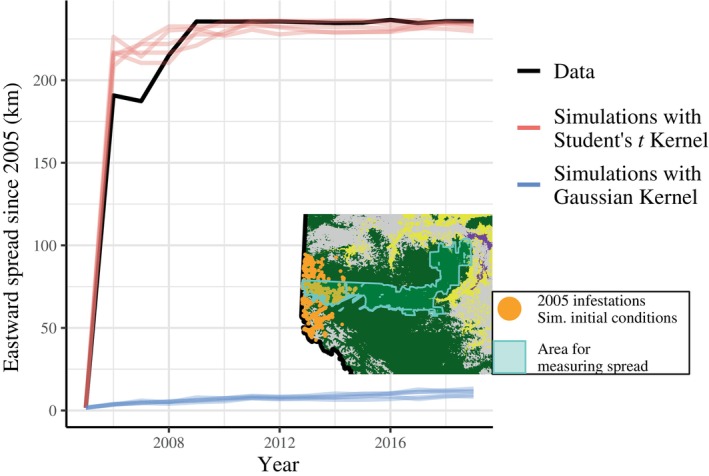
Fat‐tailed dispersal kernels can explain the spread of mountain pine beetle (MPB) across Alberta. Using the 2005 infestations as the initial condition, we simulated the spatial spread of the MPB across the lodgepole pine zone of western Alberta (the dark green area in the inset plot). To summarize the spatial extent of infestations, we calculated the maximum easting coordinate for all infestations within the cyan polygon (see inset plot). This polygon is the intersection of Heli‐GPS survey areas from 2005 to 2019.

For MPB specifically, the Student's *t*‐distribution characterized by degrees of freedom ν=1.45 and scale parameter ρ=0.0118 provides an excellent fit, and produces dispersal distances with a median, mean, and 95th percentile of 0.08, 1.04, and 4.53 km, respectively. Unlike mixture‐based dispersal kernels, the Student's *t*‐distribution faces no numerical model‐fitting problems, has only two parameters, and successfully recreates empirical patterns of distances between parental and offspring infestations (Figure 4). Although such a simple model cannot hope to capture *all* aspects of MPB dispersal, the Student's *t*‐distribution serves as a useful first‐order approximation for both researchers and conservation practitioners. Indeed, our stochastic simulations reveal that a simple fat‐tailed dispersal kernel outperforms thin‐tailed kernels in reconstructing the MPB's eastward expansion (Figure 5).

The Student's *t*‐distribution has a clear interpretation in light of MPB biology: the Student's *t*‐distribution can be derived as an infinite mixture of Fickian diffusion processes with an inverse gamma distribution for stopping times or, equivalently, for the diffusion coefficients. It is this mixture that represents the variety of MPB dispersal strategies. Three types of dispersal behaviors roughly correspond to three classes of dispersal distances: short‐range (typically <100 m), medium‐range (up to 5 km), and long‐range (above canopy, potentially up to 300 km). These distance ranges are approximate and illustrate orders of magnitude associated with each category. Short‐range dispersal predominantly occurs under the canopy, with a large majority of beetles attracted to nearby plumes of aggregation pheromones (Dodds & Ross, 2002; Robertson et al., 2007; Safranyik et al., 1992). Medium‐range dispersal involves a minority of beetles traveling up to 5 km to initiate pioneer attacks, resulting in semi‐regular patterns of so‐called spot infestations (Strohm et al., 2013). Proposed explanations for these pioneer attacks include evolutionary bet‐hedging strategies (Kautz et al., 2016; Raffa, 2001) and the absence of guiding pheromones for early‐emerging beetles. Long‐range dispersal, observed in just 0%–2.5% of beetles (Robertson et al., 2009; Safranyik et al., 1992), involves flight above the canopy, where beetles may be swept up by atmospheric winds and carried up to 300 km (Cerezke, 1989; Hiratsuka et al., 1982b; Jackson et al., 2008). Fat‐tailed dispersal kernels effectively interpolate between the three dispersal behaviors, thus capturing a wide range of dispersal distances.

Consistent with an inverse‐gamma distribution of settling times, a recent flight‐mill experiment shows that many beetles fly short distances, while few fly long distances (range 0–25 km; Evenden et al., 2014). The basis for this intraspecific variation is unclear. Larger beetles, which tend to emerge from larger trees (Graf et al., 2012), fly farther on average. However, their flight distance is also more variable with different individuals flying short or long distances (Shegelski et al., 2019). This polyphenism is thought to be an evolutionarily stable bet‐hedging strategy (Jones et al., 2020; Kautz et al., 2016), where the short‐distance dispersers are more likely to find host trees and conspecifics with which to perform mass attacks, and the risky longer distance dispersers may enjoy the fitness advantages of arriving early at a large tree (Raffa, 2001 and sources therein).

Rare, long‐distance “jackpot‐dispersal” events, as described by the tails of fat‐tailed dispersal kernels, are key for rapid spread. For example, in the case of MPB, there is evidence that their range expansion across Alberta jumped eastward 250 km in a single year (Cooke & Carroll, 2017). These events can also yield a characteristic signature pattern of patchy spread on the landscape. Such patterns are most easily seen from Monte Carlo simulations of stochastic invasion processes that have a mixture of rare long‐ and common short‐distance dispersal distances (Lewis & Pacala, 2000). The resulting spatial process, sometimes referred to as “stratified diffusion” (Shigesada et al., 1995), exhibits a characteristic patchy pattern of spread, with rare long‐distance dispersal events creating new invasion beachheads at a distance, followed by common short‐distance dispersal coupled with growth dynamics generating growth of patches on a local scale. While a full mathematical analysis of such processes typically involves spatial moments of the associated point process of invasion (Lewis & Pacala, 2000), evidence of patchy spread in the spread of MPB can be seen visually from Figure 3.

Previous research has provided a large range of dispersal estimates (recall Table 1). In particular, the work of Heavilin and Powell (2008) resulted in an implausibly small estimate of median dispersal distance: about 10 m. This result can be attributed to a least‐squares model fitting procedure, which inadequately penalizes the prediction of zero new infestations in areas where infestations occur. Our model addresses these limitations by introducing a probabilistic landscape for new infestations, such that assigning a near‐zero probability to an actual infestation incurs a near‐infinite penalty. On the other side of the spectrum, the model of Koch et al. (2021) implies an implausibly large estimate of the median dispersal distance: about 17 km. This can be explained by the two‐part structure of their model, which includes a dispersal sub‐model and a pine susceptibility sub‐model, where susceptibility is an increasing function of last year's infestations. Therefore, the short distances between successive years' infestations—normally attributed to short dispersal distance—are accounted for in the susceptibility sub‐model, leaving the dispersal parameters relatively unconstrained. Our approach allows for a more accurate estimation of dispersal distances by focusing on the redistribution of infestations rather than on individual beetles, and by taking into account inter‐annual variation in beetle productivity through the predetermined number of new infestations.

Building comprehensive models of MPB dispersal is constrained by both data availability and an incomplete understanding of key processes; we therefore prioritize models that are simple, generalizable, and immediately applicable. At small spatial scales, MPB dispersal is affected by the phenology of beetle emergence, production and diffusion of aggregation and anti‐aggregation pheromones, forest microclimates, and wind patterns (Amman & Logan, 1998; Biesinger et al., 2000). At larger spatial scales, MPB dispersal is affected by temperature patterns, windstorms, and host tree availability (Powell & Bentz, 2014; Taylor et al., 2006). Consider wind as an example of why incorporating this complexity is challenging. Wind velocities vary daily, so explaining interannual dispersal variability with wind data requires (at minimum) matching wind data to beetle flight periods; but this demands high‐resolution emergence timing data that do not currently exist. While complex models ought to achieve greater predictive accuracy for a particular location, simpler models may generalize better across different regions and time periods. Importantly, most institutions lack the time and resources to develop complex dispersal models. Our model is therefore intended as a useful, plug‐and‐play option for researchers and practitioners.

One advantage of using a dispersal kernel to model MPB is that it is relatively simple to understand and easily applied. The trade‐off is that we do not capture all biological aspects of MPB dispersal. In particular, we do not include clustering mechanisms for dispersal—for long‐distance dispersal, it would be more accurate to assume that beetles travel *en masse*, carried by wind currents—nor do we include clustering of beetles at small spatial scales to represent the effects of chemical signaling. This leads to a spatial distribution of simulated infestations that is unrealistically patchy (Appendix S1: Figure S7). Additionally, our simulations (which generate Figure 5) do not incorporate the effects of host tree depletion and assume that pine density is constant, which is roughly true over short time scales but may not be true in heavily infested areas over entire outbreaks.

Finally, beetles may disperse without necessarily infesting trees, potentially leading to underestimation of actual dispersal distances (as in Table 3). This bias arises from the Allee effect: beetles dispersing farther from infestation clusters have fewer companions for coordinating mass attacks, reducing their likelihood of successful attack and subsequent detection by Heli‐GPS surveys. However, for reasons discussed in the *Introduction* (i.e., the clumped nature of dispersal and the apparent weakness of the Allee effect), this bias may be negligible.

Our conclusions may also be particular to the study area or the years of study. We address these concerns about generalizability by considering an additional area 50 km east of the initial study area, as outlined in the *Methods*, and find qualitatively similar results (see Appendix S1). We may additionally expect inter‐annual variation in beetle dispersal given that dispersal distance may change with fluctuating environmental factors (Chen & Jackson, 2017; McCambridge, 1971; Powell & Bentz, 2014; Wijerathna and Evenden, 2020b), but note that Carroll et al. (2017) find similar distances between years. We explored inter‐annual variability by applying a Student's *t*‐dispersal kernel to each year of data independently (Appendix S1: Section S2). The median dispersal distance fluctuates with a right‐skewed distribution: most years show low distances (less than 100 m), while a few years exhibit much larger distances (several hundred meters). The variation could possibly be explained by wind patterns during MPB's dispersal phase. Wind speed affects MPB flight behavior in lab settings (Wijerathna and Evenden, 2020a), wind direction is a putative factor in MPB's dispersal across the Canadian Rocky Mountains (Chen & Jackson, 2017; Jackson et al., 2008), and atmospheric models can predict long‐distance dispersal in other forest pests (e.g., the spruce budworm, Sturtevant et al., 2013). However, there is a dearth of studies relating wind to under‐canopy and in situ MPB dispersal (but see Safranyik et al., 1989).

Models that approximate stratified diffusion are particularly valuable because the pattern of numerous short‐distance dispersers complemented by relatively few long‐distance “jumpers” appears to be a common feature of successful biological invasions across diverse taxa (Mineur et al., 2010; Suarez et al., 2001; Ward et al., 2020). This pattern enables species to both exploit local resources through short dispersal and discover distant suitable habitats through long‐distance dispersal events, potentially allowing them to bypass unsuitable habitat. Intraspecific variability in dispersal distance is an evolutionarily stable strategy in spatiotemporally fluctuating environments (Dieckmann et al., 1999; McPeek & Holt, 1992). Fat‐tailed dispersal kernels effectively model this common pattern, though validation across additional species and geographic regions is needed to establish them as the preferred class of models for forest pest dispersal. A fundamental challenge in extending our methodology lies in distinguishing infestations caused by immigration versus those caused by the irruption of previously unobserved endemic populations.

The Student's *t*‐distribution serves as a practical tool for forest managers and researchers studying forest pests. With only two parameters, it can be estimated using aerial survey data while capturing a wide range of dispersal behaviors—the distribution interpolates between exclusively short‐distance dispersal and stratified dispersal. While we have used Heli‐GPS surveys, the gold standard for remotely sensing forest pests (Nelson et al., 2006), it is possible that airplane‐based aerial surveys could also be used; importantly, these data are publicly available for the majority of forested land throughout the western United States and Canada (Government of Alberta, 2016; Johnson, 2016; Johnson & Wittwer, 2006). In the case of MPB, we have shown that long‐distance dispersal events are key for describing range expansion, pointing to the importance of simple but accurate dispersal kernels for understanding ecological processes.

## AUTHOR CONTRIBUTIONS

All authors conceived the project; Evan C. Johnson performed the analysis; Evan C. Johnson and Micah Brush wrote the first draft; all authors contributed critically to the drafts and gave final approval for publication.

## CONFLICT OF INTEREST STATEMENT

The authors declare no conflicts of interest.

## Supporting information


Appendix S1.

## Data Availability

Data and code (Johnson, 2025) are available in Figshare at https://doi.org/10.6084/m9.figshare.30644852.v1. Some data supporting this research are restricted and not available publicly. Heli‐GPS data for mountain pine beetle infestations are owned by the provincial government of Alberta and may be made available to qualified researchers by contacting Mike Undershulz, Senior Forest Entomologist in the Alberta Forestry and Parks department (Mike.Undershultz@gov.ab.ca), and requesting Heli‐GPS data.

## References

[ecy70305-bib-0001] Allen, L. J. 2010. An Introduction to Stochastic Processes with Applications to Biology. Boca Raton, FL: CRC Press.

[ecy70305-bib-0002] Amman, G. D. , and J. A. Logan . 1998. “Silvicultural Control of Mountain Pine Beetle: Prescriptions and the Influence of Microclimate.” American Entomologist 44(3): 166–178.

[ecy70305-bib-0003] Anderson, D. P. , and B. R. Sturtevant . 2011. “Pattern Analysis of Eastern Spruce Budworm Choristoneura Fumiferana Dispersal.” Ecography 34(3): 488–497.

[ecy70305-bib-0004] Aukema, B. H. , A. L. Carroll , Y. Zheng , J. Zhu , K. F. Raffa , R. Dan Moore , K. Stahl , and S. W. Taylor . 2008. “Movement of Outbreak Populations of Mountain Pine Beetle: Influences of Spatiotemporal Patterns and Climate.” Ecography 31(3): 348–358.

[ecy70305-bib-0005] Benhamou, S. 2007. “How Many Animals Really Do the Lévy Walk?” Ecology 88(8): 1962–1969. 17824427 10.1890/06-1769.1

[ecy70305-bib-0006] Bentz, B. J. , J. A. Powell , and J. A. Logan . 1996. “Localized Spatial and Temporal Attack Dynamics of the Mountain Pine Beetle in Lodgepole Pine.” Forest Service research paper. Technical Report PB‐97‐129043/XAB; FSRP/INT‐494, Forest Service, Ogden, UT (United States). Intermountain Research Station.

[ecy70305-bib-0007] Biesinger, Z. , J. Powell , B. Bentz , and J. A. Logan . 2000. “Direct and Indirect Parametrization of a Localized Model for the Mountain Pine Beetle — Lodgepole Pine System.” Ecological Modelling 129(2): 273–296.

[ecy70305-bib-0008] Bleiker, K. P. , ed. 2019. Risk Assessment of the Threat of Mountain Pine Beetle to Canada's Boreal and Eastern Pine Forests: Prepared for the Canadian Council of Forest Ministers, Forest Pest Working Group. Victoria: Canadian Council of Forest Ministers.

[ecy70305-bib-0009] Bleiker, K. P. , M. R. O'Brien , G. D. Smith , and A. L. Carroll . 2014. “Characterisation of Attacks Made by the Mountain Pine Beetle (Coleoptera: Curculionidae) during its Endemic Population Phase.” The Canadian Entomologist 146(3): 271–284.

[ecy70305-bib-0010] Boone, C. K. , B. H. Aukema , J. Bohlmann , A. L. Carroll , and K. F. Raffa . 2011. “Efficacy of Tree Defense Physiology Varies with Bark Beetle Population Density: A Basis for Positive Feedback in Eruptive Species.” Canadian Journal of Forest Research 41(6): 1174–1188.

[ecy70305-bib-0011] Broadbent, S. R. , and D. G. Kendall . 1953. “The Random Walk of *Trichostrongylus retortaeformis* .” Biometrics 9(4): 460.

[ecy70305-bib-0012] Capinera, J. L. , and P. Barbosa . 1976. “Dispersal of First‐Instar Gypsy Moth Larvae in Relation to Population Quality.” Oecologia 26: 53–60. 28309103 10.1007/BF00345652

[ecy70305-bib-0013] Carroll, A. , J. Régnière , J. Logan , S. Taylor , B. Bentz , and J. Powell . 2006. “Impacts of Climate Change on Range Expansion by the Mountain Pine Beetle.” Technical Report, Pacific Forestry Centre Canada.

[ecy70305-bib-0014] Carroll, A. , B. Seely , C. Welham , and H. Nelson . 2017. “Assessing the Effectiveness of Alberta's Forest Management Program Against the Mountain Pine Beetle: Final Report for fRI Research Project 246.18 Parts 1 and 2.” Technical Report, fRI Research.

[ecy70305-bib-0015] Carroll, A. L. , B. Aukema , K. Raffa , D. Linton , G. D. Smith , and B. Lindgren . 2006. “Mountain Pine Beetle Outbreak Development: The Endemic—Incipient Epidemic Transition.” Mpbi Project # 1.03, Natural Resources Canada, Canadian Forest Service.

[ecy70305-bib-0016] Carroll, A. L. , and L. Safranyik . 2003. “The Bionomics of the Mountain Pine Beetle in Lodgepole Pine Forests: Establishing a Context.” In Mountain pine beetle symposium: Challenges and solutions, pp. 21–32. Natural Resources Canada, Canadian Forest Service

[ecy70305-bib-0017] CBC News . 2023. “Mountain Pine Beetle Populations Down by 94 per cent in Alberta Since 2019: Province.”

[ecy70305-bib-0018] Cerezke, H. F. 1989. “Mountain Pine Beetle Aggregation Semiochemical Use in Alberta and Saskatchewan, 1983–1987.” General Technical Report INT‐262, United States Department of Agriculture, Forest Service, Ogden, Utah, Kalispell, Montana.

[ecy70305-bib-0019] Chen, H. , and P. L. Jackson . 2017. “Climatic Conditions for Emergence and Flight of Mountain Pine Beetle: Implications for Long‐Distance Dispersal.” Canadian Journal of Forest Research 47(7): 974–984.

[ecy70305-bib-0020] Cooke, B. , C. MacQuarrie , and A. Carroll . 2024. “On the Deduction and Quantification of Irruptive Dynamics in Mountain Pine Beetle Population and Proxy Data.” Journal of Applied Entomology 149(3): 309–323.

[ecy70305-bib-0021] Cooke, B. J. , and A. L. Carroll . 2017. “Predicting the Risk of Mountain Pine Beetle Spread to Eastern Pine Forests: Considering Uncertainty in Uncertain Times.” Forest Ecology and Management 396: 11–25.

[ecy70305-bib-0022] Corbett, L. J. , P. Withey , V. Lantz , and T. Ochuodho . 2016. “The Economic Impact of the Mountain Pine Beetle Infestation in British Columbia: Provincial Estimates from a CGE Analysis.” Forestry: An International Journal of Forest Research 89(1): 100–105.

[ecy70305-bib-0023] Creeden, E. P. , J. A. Hicke , and P. C. Buotte . 2014. “Climate, Weather, and Recent Mountain Pine Beetle Outbreaks in the Western United States.” Forest Ecology and Management 312: 239–251.

[ecy70305-bib-0024] Cudmore, T. J. , N. Björklund , A. L. Carroll , and S. B. Lindgren . 2010. “Climate Change and Range Expansion of an Aggressive Bark Beetle: Evidence of Higher Beetle Reproduction in naïve Host Tree Populations.” Journal of Applied Ecology 47(5): 1036–1043.

[ecy70305-bib-0025] Dieckmann, U. , B. O'Hara , and W. Weisser . 1999. “The Evolutionary Ecology of Dispersal.” Trends in Ecology & Evolution 14(3): 88–90.

[ecy70305-bib-0026] Dodds, K. J. , and D. W. Ross . 2002. “Sampling Range and Range of Attraction of *Dendroctonus pseudotsugae* Pheromone‐Baited Traps.” The Canadian Entomologist 134(3): 343–355.

[ecy70305-bib-0027] Doležal, P. , J. Okrouhlik , and M. Davidkova . 2016. “Fine Fluorescent Powder Marking Study of Dispersal in the Spruce Bark Beetle, *Ips typographus* (Coleoptera: Scolytidae).” European Journal of Entomology 113: 1–8.

[ecy70305-bib-0028] Evenden, M. L. , C. M. Whitehouse , and J. Sykes . 2014. “Factors Influencing Flight Capacity of the Mountain Pine Beetle (Coleoptera: Curculionidae: Scolytinae).” Environmental Entomology 43(1): 187–196. 24367930 10.1603/EN13244

[ecy70305-bib-0029] Faccoli, M. , and F. Stergulc . 2004. “ *Ips typographus* (L.) Pheromone Trapping in South Alps: Spring Catches Determine Damage Thresholds.” Journal of Applied Entomology 128(4): 307–311.

[ecy70305-bib-0030] Garcia, M. , B. R. Sturtevant , R. Saint‐Amant , J. J. Charney , J. Delisle , Y. Boulanger , P. A. Townsend , and J. Régnière . 2022. “Modeling Weather‐Driven Long‐Distance Dispersal of Spruce Budworm Moths (*Choristoneura fumiferana*). Part 1: Model Description.” Agricultural and Forest Meteorology 315: 108815.

[ecy70305-bib-0031] Goodsman, D. W. , D. Koch , C. Whitehouse , M. L. Evenden , B. J. Cooke , and M. A. Lewis . 2016. “Aggregation and a Strong Allee Effect in a Cooperative Outbreak Insect.” Ecological Applications 26(8): 2623–2636. 10.1002/eap.1404 27862568

[ecy70305-bib-0032] Government of Alberta . 2016. “Mountain Pine Beetle Detection and Management in Alberta.” Technical Report, Government of Alberta, Agriculture and Forestry.

[ecy70305-bib-0033] Graf, M. , M. Reid , B. Aukema , and B. Lindgren . 2012. “Association of Tree Diameter with Body Size and Lipid Content of Mountain Pine Beetles.” The Canadian Entomologist 144(3): 467–477.

[ecy70305-bib-0034] Heavilin, J. , and J. A. Powell . 2008. “A Novel Method of Fitting Spatio‐Temporal Models to Data, with Applications to the Dynamics of Mountain Pine Beetles.” Natural Resource Modeling 21(4): 489–524.

[ecy70305-bib-0035] Hiratsuka, Y. , H. F. Cerezke , B. H. Moody , J. Petty , and G. N. Still . 1982a. Forest Insect and Disease Conditions in Alberta, Saskatchewan, Manitoba, and the Northwest Territories in 1981 and Predictions for 1982. Information Report NOR‐X‐239. Edmonton, AB: Natural Resources Canada, Canadian Forest Service.

[ecy70305-bib-0036] Hiratsuka, Y. , H. F. Cerezke , B. H. Moody , J. A. Petty , and G. N. Still . 1982b. Forest Insect and Disease Conditions in Alberta, Saskatchewan, Manitoba, and the Northwest Territories in 1981 and Predictions for 1982. Information report NOR‐X‐239. Edmonton, AB: Environment Canada, Canadian Forestry Service, Northern Forest Research Centre.

[ecy70305-bib-0037] Howe, M. , A. Carroll , C. Gratton , and K. F. Raffa . 2021. “Climate‐Induced Outbreaks in High‐Elevation Pines Are Driven Primarily by Immigration of Bark Beetles from Historical Hosts.” Global Change Biology 27(22): 5786–5805. 34428326 10.1111/gcb.15861

[ecy70305-bib-0038] Jackson, P. L. , D. Straussfogel , B. S. Lindgren , S. Mitchell , and B. Murphy . 2008. “Radar Observation and Aerial Capture of Mountain Pine Beetle, *Dendroctonus ponderosae* Hopk. (Coleoptera: Scolytidae) in Flight above the Forest Canopy.” Canadian Journal of Forest Research 38(8): 2313–2327.

[ecy70305-bib-0039] Johnson, D. M. , O. N. Bjørnstad , and A. M. Liebhold . 2004. “Landscape Geometry and Travelling Waves in the Larch Budmoth.” Ecology Letters 7(10): 967–974.

[ecy70305-bib-0040] Johnson, D. M. , A. M. Liebhold , P. C. Tobin , and O. N. Bjørnstad . 2006. “Allee Effects and Pulsed Invasion by the Gypsy Moth.” Nature 444(7117): 361–363. 17108964 10.1038/nature05242

[ecy70305-bib-0041] Johnson, E. 2025. “Data and Code for ‘Modeling Stratified Dispersal in Forest Pests: A Case Study of the Mountain Pine Beetle in Alberta.” Figshare. 10.6084/m9.figshare.30644852.v1. PMC12866754 41631857

[ecy70305-bib-0042] Johnson, E. W. , and D. Wittwer . 2006. “Aerial Detection Surveys in the United States.” Technical Report RMRS‐P‐42CD, USDA Forest Service, Rocky Mountain Research Station, Fort Collins, CO. Proceedings of the Forest Health Monitoring Program.

[ecy70305-bib-0043] Johnson, J. 2016. “Aerial Forest Insect and Disease Detection Surveys in Oregon and Washington 1947–2016 – the Survey.” Technical Report, United States Department of Agriculture, Forest Service.

[ecy70305-bib-0044] Jones, K. L. , R. Rajabzadeh , G. Ishangulyyeva , N. Erbilgin , and M. L. Evenden . 2020. “Mechanisms and Consequences of Flight Polyphenisms in an Outbreaking Bark Beetle Species.” Journal of Experimental Biology 223(12): jeb219642. 32341173 10.1242/jeb.219642

[ecy70305-bib-0045] Jones, K. L. , V. A. Shegelski , N. G. Marculis , A. N. Wijerathna , and M. L. Evenden . 2019. “Factors Influencing Dispersal by Flight in Bark Beetles (Coleoptera: Curculionidae: Scolytinae): From Genes to Landscapes.” Canadian Journal of Forest Research 49(9): 1024–1041.

[ecy70305-bib-0046] Joseph, J. , and H. Sendner . 1958. “Über die horizontale Diffusion im Meere.” Deutsche Hydrographische Zeitschrift 11(2): 49–77.

[ecy70305-bib-0047] Kautz, M. , K. Dworschak , A. Gruppe , and R. Schopf . 2011. “Quantifying Spatio‐Temporal Dispersion of Bark Beetle Infestations in Epidemic and Non‐epidemic Conditions.” Forest Ecology and Management 262(4): 598–608.

[ecy70305-bib-0048] Kautz, M. , M. A. Imron , K. Dworschak , and R. Schopf . 2016. “Dispersal Variability and Associated Population‐Level Consequences in Tree‐Killing Bark Beetles.” Movement Ecology 4(1): 9. 27087978 10.1186/s40462-016-0074-9 PMC4832482

[ecy70305-bib-0049] Koch, D. , M. A. Lewis , and S. Lele . 2021. “The Signature of Endemic Populations in the Spread of Mountain Pine Beetle Outbreaks.” Bulletin of Mathematical Biology 83(6): 65. 33932176 10.1007/s11538-021-00899-z

[ecy70305-bib-0050] Koch, D. C. , M. A. Lewis , and S. R. Lele . 2020. “A Unifying Theory for Two‐Dimensional Spatial Redistribution Kernels with Applications in Population Spread Modelling.” Journal of the Royal Society Interface 17(170): 20200434. 32993427 10.1098/rsif.2020.0434 PMC7536060

[ecy70305-bib-0051] Kot, M. , M. A. Lewis , and P. van den Driessche . 1996. “Dispersal Data and the Spread of Invading Organisms.” Ecology 77(7): 2027–2042.

[ecy70305-bib-0052] Lewis, M. A. , and S. Pacala . 2000. “Modeling and Analysis of Stochastic Invasion Processes.” Journal of Mathematical Biology 41: 387–429. 11151706 10.1007/s002850000050

[ecy70305-bib-0053] Lewis, M. A. , S. Petrovskii , and J. Potts . 2016. The Mathematics behind Biological Invasions. New York, NY: Springer Berlin Heidelberg.

[ecy70305-bib-0054] Liu, B. R. , and M. Kot . 2019. “Accelerating Invasions and the Asymptotics of Fat‐Tailed Dispersal.” Journal of Theoretical Biology 471: 22–41. 30914297 10.1016/j.jtbi.2019.03.016

[ecy70305-bib-0055] McCambridge, W. F. 1971. “Temperature Limits of Flight of the Mountain Pine Beetle, *Dendroctonus ponderosae* .” Annals of the Entomological Society of America 64(2): 534–535.

[ecy70305-bib-0056] McPeek, M. A. , and R. D. Holt . 1992. “The Evolution of Dispersal in Spatially and Temporally Varying Environments.” The American Naturalist 140(6): 1010–1027.

[ecy70305-bib-0057] Mineur, F. , A. J. Davies , C. A. Maggs , M. Verlaque , and M. P. Johnson . 2010. “Fronts, Jumps and Secondary Introductions Suggested as Different Invasion Patterns in Marine Species, with an Increase in Spread Rates over Time.” Proceedings of the Royal Society B: Biological Sciences 277(1694): 2693–2701. 10.1098/rspb.2010.0494 PMC2982048 20410039

[ecy70305-bib-0058] Muirhead, J. R. , B. Leung , C. van Overdijk , D. W. Kelly , K. Nandakumar , K. R. Marchant , and H. J. MacIsaac . 2006. “Modelling Local and Long‐Distance Dispersal of Invasive Emerald Ash Borer *Agrilus planipennis* (Coleoptera) in North America.” Diversity and Distributions 12(1): 71–79.

[ecy70305-bib-0059] Nealis, V. G. , and B. J. Cooke . 2014. “Risk Assessment of the Threat of Mountain Pine Beetle to Canada's Boreal and Eastern Pine Forests.” Technical Report, Natural Resources Canada, Canadian Forest Service, Pacific Forestry Centre.

[ecy70305-bib-0060] Nelson, T. , B. Boots , and M. A. Wulder . 2006. “Large‐Area Mountain Pine Beetle Infestations: Spatial Data Representation and Accuracy.” The Forestry Chronicle 82(2): 243–252.

[ecy70305-bib-0061] Peltonen, M. , A. M. Liebhold , O. N. Bjørnstad , and D. W. Williams . 2002. “Spatial Synchrony in Forest Insect Outbreaks: Roles of Regional Stochasticity and Dispersal.” Ecology 83(11): 3120–3129.

[ecy70305-bib-0062] Powell, J. A. , and B. J. Bentz . 2014. “Phenology and Density‐Dependent Dispersal Predict Patterns of Mountain Pine Beetle (*Dendroctonus ponderosae*) Impact.” Ecological Modelling 273: 173–185.

[ecy70305-bib-0063] Preisler, H. K. , J. A. Hicke , A. A. Ager , and J. L. Hayes . 2012. “Climate and Weather Influences on Spatial Temporal Patterns of Mountain Pine Beetle Populations in Washington and Oregon.” Ecology 93(11): 2421–2434. 23236913 10.1890/11-1412.1

[ecy70305-bib-0064] R Core Team . 2022. R: A Language and Environment for Statistical Computing. Vienna: R Foundation for Statistical Computing.

[ecy70305-bib-0065] Raffa, K. F. 2001. “Mixed Messages across Multiple Trophic Levels: The Ecology of Bark Beetle Chemical Communication Systems.” Chemoecology 11(2): 49–65.

[ecy70305-bib-0066] Raffa, K. F. , and A. A. Berryman . 1983. “The Role of Host Plant Resistance in the Colonization Behavior and Ecology of Bark Beetles (Coleoptera: Scolytidae).” Ecological Monographs 53(1): 27–49.

[ecy70305-bib-0067] Régnière, J. , J. Delisle , D. S. Pureswaran , and R. Trudel . 2013. “Mate‐Finding Allee Effect in Spruce Budworm Population Dynamics.” Entomologia Experimentalis et Applicata 146(1): 112–122.

[ecy70305-bib-0068] Rieske, L. , and L. Townsend . 2005. “Orientation and Dispersal Patterns of the Eastern Tent Caterpillar, *Malacosoma americanum* F. (Lepidoptera: Lasiocampidae).” Journal of Insect Behavior 18: 193–207.

[ecy70305-bib-0069] Robertson, C. , T. A. Nelson , and B. Boots . 2007. “Mountain Pine Beetle Dispersal: The Spatial‐Temporal Interaction of Infestations.” Forest Science 53(3): 395–405.

[ecy70305-bib-0070] Robertson, C. , T. A. Nelson , D. E. Jelinski , M. A. Wulder , and B. Boots . 2009. “Spatial–Temporal Analysis of Species Range Expansion: The Case of the Mountain Pine Beetle, *Dendroctonus ponderosae* .” Journal of Biogeography 36(8): 1446–1458.

[ecy70305-bib-0071] Rogers, H. S. , N. G. Beckman , F. Hartig , J. S. Johnson , G. Pufal , K. Shea , D. Zurell , et al. 2019. “The Total Dispersal Kernel: A Review and Future Directions.” AoB Plants 11(5): plz042. 31579119 10.1093/aobpla/plz042 PMC6757349

[ecy70305-bib-0072] Safranyik, L. , and A. L. Carroll . 2006. “The Biology and Epidemiology of the Mountain Pine Beetle in Lodgepole Pine Forests.” In The Mountain Pine Beetle: A Synthesis of Biology, Management, and Impacts on Lodgepole Pine, edited by L. Safranyik and B. Wilson , 3–66. Victoria, BC: Natural Resources Canada, Canadian Forest Service, Pacific Forestry Centre.

[ecy70305-bib-0073] Safranyik, L. , D. A. Linton , R. Silversides , and L. H. McMullen . 1992. “Dispersal of Released Mountain Pine Beetles under the Canopy of a Mature Lodgepole Pine Stand.” Journal of Applied Entomology 113(1–5): 441–450.

[ecy70305-bib-0074] Safranyik, L. , R. Silversides , L. H. McMullen , and D. A. Linton . 1989. “An Empirical Approach to Modeling the Local Dispersal of the Mountain Pine Beetle (*Dendroctonus ponderosae* Hopk.) (Col., Scolytidae) in Relation to Sources of Attraction, Wind Direction and Speed.” Journal of Applied Entomology 108(1–5): 498–511.

[ecy70305-bib-0075] Sambaraju, K. R. , A. L. Carroll , J. Zhu , K. Stahl , R. D. Moore , and B. H. Aukema . 2012. “Climate Change Could Alter the Distribution of Mountain Pine Beetle Outbreaks in Western Canada.” Ecography 35(3): 211–223.

[ecy70305-bib-0076] Schupp, E. W. , P. Jordano , and J. M. Gómez . 2010. “Seed Dispersal Effectiveness Revisited: A Conceptual Review.” New Phytologist 188(2): 333–353. 20673283 10.1111/j.1469-8137.2010.03402.x

[ecy70305-bib-0077] Shegelski, V. A. , M. L. Evenden , and F. A. H. Sperling . 2019. “Morphological Variation Associated with Dispersal Capacity in a Tree‐Killing Bark Beetle *Dendroctonus ponderosae* Hopkins.” Agricultural and Forest Entomology 21(1): 79–87.

[ecy70305-bib-0078] Shigesada, N. , K. Kawasaki , and Y. Takeda . 1995. “Modeling Stratified Diffusion in Biological Invasions.” American Naturalist 146(2): 229–251.

[ecy70305-bib-0079] Simard, M. , E. N. Powell , K. F. Raffa , and M. G. Turner . 2012. “What Explains Landscape Patterns of Tree Mortality Caused by Bark Beetle Outbreaks in Greater Yellowstone?” Global Ecology and Biogeography 21(5): 556–567.

[ecy70305-bib-0080] Six, D. L. , C. Vergobbi , and M. Cutter . 2018. “Are Survivors Different? Genetic‐Based Selection of Trees by Mountain Pine Beetle during a Climate Change‐Driven Outbreak in a High‐Elevation Pine Forest.” Frontiers in Plant Science 9: 993. 30083173 10.3389/fpls.2018.00993 PMC6064936

[ecy70305-bib-0081] Skellam, J. G. 1951. “Random Dispersal in Theoretical Populations.” Biometrika 38(1/2): 196. 14848123

[ecy70305-bib-0082] Srivastava, V. , and A. L. Carroll . 2023. “Dynamic Distribution Modelling Using a Native Invasive Species, the Mountain Pine Beetle.” Ecological Modelling 482: 110409.

[ecy70305-bib-0083] Strohm, S. , R. C. Tyson , and J. A. Powell . 2013. “Pattern Formation in a Model for Mountain Pine Beetle Dispersal: Linking Model Predictions to Data.” Bulletin of Mathematical Biology 75(10): 1778–1797. 23925726 10.1007/s11538-013-9868-8

[ecy70305-bib-0084] Sturtevant, B. R. , G. L. Achtemeier , J. J. Charney , D. P. Anderson , B. J. Cooke , and P. A. Townsend . 2013. “Long‐Distance Dispersal of Spruce Budworm (*Choristoneura fumiferana* Clemens) in Minnesota (USA) and Ontario (Canada) Via the Atmospheric Pathway.” Agricultural and Forest Meteorology 168: 186–200.

[ecy70305-bib-0085] Suarez, A. V. , D. A. Holway , and T. J. Case . 2001. “Patterns of Spread in Biological Invasions Dominated by Long‐Distance Jump Dispersal: Insights from Argentine Ants.” Proceedings of the National Academy of Sciences 98(3): 1095–1100. 10.1073/pnas.98.3.1095 PMC14714 11158600

[ecy70305-bib-0086] Taylor, S. W. , A. L. Carroll , R. I. Alfaro , and L. Safranyik . 2006. “Forest, Climate and Mountain Pine Beetle Outbreak Dynamics in Western Canada.” In The Mountain Pine Beetle: A Synthesis of Biology, Management, and Impacts on Lodgepole Pine, edited by L. Safranyik and B. Wilson . Victoria, BC: Natural Resources Canada, Canadian Forest Service, Pacific Forestry Centre.

[ecy70305-bib-0087] Turchin, P. , and W. T. Thoeny . 1993. “Quantifying Dispersal of Southern Pine Beetles with Mark‐Recapture Experiments and a Diffusion Model.” Ecological Applications 3(1): 187–198. 27759232 10.2307/1941801

[ecy70305-bib-0088] Walton, A. 2012. “Provincial‐Level Projection of the Current Mountain Pine Beetle Outbreak: Update of the Infestation Projection Based on the Provincial Aerial Overview Surveys of Forest Health Conducted from 1999 Through 2011 and the BCMPB Model (Year 9).” Technical Report, BC Forest Service.

[ecy70305-bib-0089] Ward, S. F. , S. Fei , and A. M. Liebhold . 2020. “Temporal Dynamics and Drivers of Landscape‐Level Spread by Emerald Ash Borer.” Journal of Applied Ecology 57(6): 1020–1030.

[ecy70305-bib-0090] Werner, R. A. , and E. H. Holsten . 1997. “Dispersal of the Spruce Beetle, *Dendroctonus rufipennis*, and the Engraver Beetle, *Ips perturbatus*, in Alaska.” Research Paper PNW‐RP‐501, U.S. Department of Agriculture, Forest Service, Pacific Northwest Research Station, Portland, OR.

[ecy70305-bib-0091] Wijerathna, A. , and M. Evenden . 2020. “Effect of Environmental Conditions on Flight Capacity in Mountain Pine Beetle (Coleoptera: Curculionidae: Scolytinae).” Journal of Insect Behavior 33(5): 201–215.

[ecy70305-bib-0092] Withrow, J. R. , J. E. Lundquist , and J. F. Negrón . 2013. “Spatial Dispersal of Douglas‐Fir Beetle Populations in Colorado and Wyoming.” ISRN Forestry 2013: 1–10.

[ecy70305-bib-0093] Zolubas, P. , and J. A. Byers . 1995. “Recapture of Dispersing Bark Beetle *Ips typographus* L. (*Col*., *Scolytidae*) in Pheromone‐Baited Traps: Regression Models.” Journal of Applied Entomology 119(1–5): 285–289.

[ecy70305-bib-0094] Zumr, V. 1992. “Dispersal of the Spruce Bark Beetle *Ips typographus* (L.) (*Col*., *Scolytidae*) in Spruce Woods.” Journal of Applied Entomology 114(1–5): 348–352.

