# Supplementary material for: Modeling stratified dispersal in forest pests: A case study of the mountain pine beetle in Alberta
Source: Ecology. 2026 Feb 3;107(2):e70305. doi: 10.1002/ecy.70305 (PMC12866754; doi:10.1002/ecy.70305)
Supplement: Supplementary file 1 — Appendix S1. [file ECY-107-e70305-s001.pdf]

Appendix S1

Supporting information for “Modeling stratified dispersal in forest pests: A case study of the mountain pine beetle in Alberta”

Evan C. Johnson, Micah Brush, and Mark A. Lewis

Contents

S1 Robustness check: Study area #2 **2**

S2 Robustness check: inter-annual dispersal variability **4**

S3 Additional information about characteristic length scales **5**

S4 Additional figures & tables 8

Section S1 Robustness check: Study area #2

The nature of MPB dispersal may vary across space, possibly due to unmodeled factors such as MPB population density, pine density, wind patterns, and annual temperature patterns. As reassurance that our dispersal models will produce reasonable results, even if applied outside of our focal study area, we re-ran our analysis in a second study area. This area is located approximately 50 km east of study area #1 (Fig. S1) and has approximate dimensions of 50x50 km.

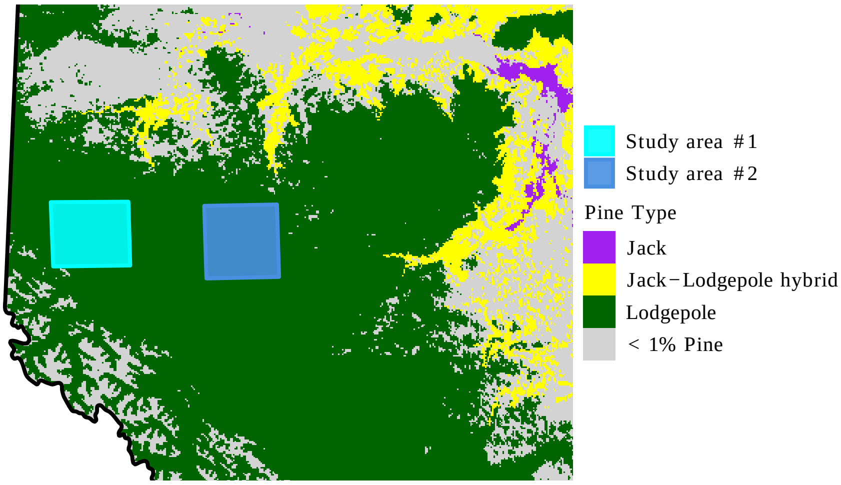

Figure S1: The location and extent of both study areas in Alberta.

In study area #2, the estimated median and mean dispersal distances for the Student's t kernel are 0.060 and 0.988 km respectively (Table S1). These numbers are extremely similar to the estimates from study area #1 (Table 3). In study area #2, fat-tailed dispersal kernels are superior to mixture-based dispersal kernels with respect to the log likelihood; the true positive rate of predicting new infestations; and the correlation between log-scale predictions and infestations. For completeness, parameter estimates for study area #2 are provided in Table S2.

Table S1: Summary statistics of model fit and redistribution distances for the redistribution models, fit to data from study area # 2. The abbreviation TPR stands for *True Positive Rate*; r is the correlation between the logarithm of the observed number of infestations and the logarithm of the expected number of infestations; and the last 4 columns refer to the distances between new infestations and their parental infestations.

Kernel name	Log likelihood	TPR	r (log scale)	mean dist.	median dist. (km)	75% dist. (km)	95% dist. (km)
Pareto	$-1.491 \cdot 10^6$	0.094	0.299	0.820	0.073	0.270	3.162
Student's t	$-1.491 \cdot 10^6$	0.108	0.295	0.967	0.058	0.238	4.059
Bessel mixture	$-1.499 \cdot 10^6$	0.082	0.298	0.530	0.071	0.771	2.343
Laplace mixture	$-1.504 \cdot 10^6$	0.097	0.289	0.486	0.073	0.790	1.884
Gaussian mixture	$-1.518 \cdot 10^6$	0.106	0.274	0.699	0.469	1.249	2.161
WMY	$-1.582 \cdot 10^6$	0.000	0.241	0.307	0.246	0.418	0.781
Bessel	$-1.582 \cdot 10^6$	0.000	0.246	0.337	0.270	0.459	0.859
Laplace	$-1.605 \cdot 10^6$	0.000	0.231	0.350	0.294	0.471	0.830
Gaussian	$-1.657 \cdot 10^6$	0.000	0.194	0.652	0.612	0.866	1.272

Table S2: Parameter values for the redistribution models, fit to data from study area #2. The parameters ρ, ρ_1, and ρ_2 have units of kilometers; the remaining parameters are dimensionless.

Kernel name	Tail type	$D(r) \propto$	Max likelihood estimate
Pareto	Fat-tail	$(r+\rho)^{-(1+\nu)}$	$\rho = 1.45 \cdot 10^{-2},\ \nu = 1.61$
Student's t	Fat-tail	$\left(1+\frac{1}{\nu}\left(\frac{r}{\rho}\right)^2\right)^{-\frac{\nu+1}{2}}$	$\rho = 1.16 \cdot 10^{-2},\ \nu = 1.47$
Bessel mixture	Mixture of Thin	$\theta\, K_0(\frac{r}{\rho_1}) + (1-\theta)K_0(\frac{r}{\rho_2})$	$\rho_1 = 2.65 \cdot 10^{-2},\ \rho_2 = 7.84 \cdot 10^{-1},\ \theta = 5.90 \cdot 10^{-1}$
Laplace mixture	Mixture of Thin	$\theta \exp\left(-\frac{r}{\rho_1}\right) + (1-\theta)\exp\left(-\frac{r}{\rho_2}\right)$	$\rho_1 = 1.55 \cdot 10^{-2},\ \rho_2 = 4.91 \cdot 10^{-1},\ \theta = 5.22 \cdot 10^{-1}$
Gaussian mixture	Mixture of Thin	$\theta \exp\left(-\left(\frac{r}{\rho_1}\right)^2\right) + (1-\theta)\exp\left(-\left(\frac{r}{\rho_2}\right)^2\right)$	$\rho_1 = 2.23 \cdot 10^{-2},\ \rho_2 = 1.39,\ \theta = 4.40 \cdot 10^{-1}$
WMY	Thin tail	$\left(\frac{r}{\rho}\right)^{\kappa} K_\kappa(\frac{r}{\rho})$	$\rho = 1.95 \cdot 10^{-1},\ \kappa = 2.96 \cdot 10^{-8}$
Bessel	Thin tail	$K_0(\frac{r}{\rho})$	$\rho = 2.15 \cdot 10^{-1}$
Laplace	Thin tail	$\exp\left(-\frac{r}{\rho}\right)$	$\rho = 1.75 \cdot 10^{-1}$
Gaussian	Thin tail	$\exp\left(-\left(\frac{r}{\rho}\right)^2\right)$	$\rho = 7.35 \cdot 10^{-1}$

Section S2 Robustness check: inter-annual dispersal variability

Just as dispersal may exhibit spatial heterogeneity, it may also exhibit temporal heterogeneity. To examine this, we applied a Student's t dispersal kernel to each year's data independently. The resulting estimates reveal that the median dispersal distance fluctuates over time, although generally within narrow bounds. Specifically, while the median typically stays in the range of 0.02 – 0.1 km (centered around 0.05 km), there were 2/22 estimates which exceeded 0.3 km (Fig. S2).

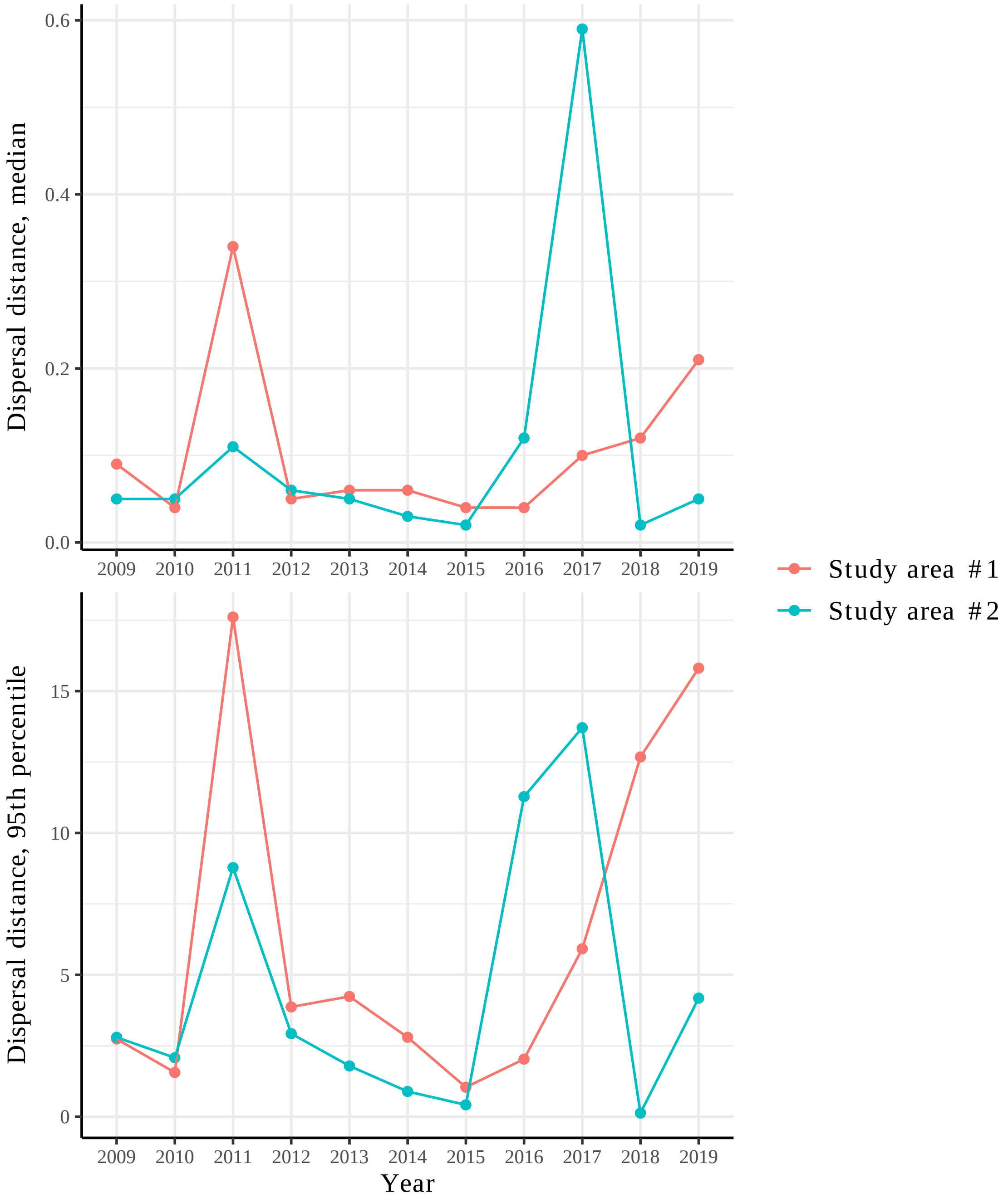

Figure S2: Estimates of dispersal distance across years, for both study areas.

Section S3 Additional information about characteristic length scales

Table 1 shows that the typical length scale of MPB dispersal varies widely across the literature, as well as data from related bark beetles. Here, in Table S3, we show in more detail where we obtained the stated values in that table for MPB specifically, and in Table S4 we show more detail for the stated values for related bark beetles.

Table S3: Additional information on the stated values in Table 1 for mountain pine beetle. This includes information about the study location as well as more details on where we obtained the characteristic scale of infestations.

Study	Scale (m)	Study location	Source of scale
Aukema et al. (2008)	18 000	British Columbia	Finds that the neighborhood of one cell is the most important predictor for infestations in 12 km x 12 km cells, which corresponds to roughly 18 km
Koch et al. (2021)	17 000	British Columbia	Finds 17 km for the median dispersal distance from fitting an anisotropic WMY dispersal kernel to infestation data (*personal communication*)
Preisler et al. (2012)	10 000	Washington and Oregon	Finds that a distance weighted beetle pressure metric out to 10 km is one of the most important variables for all stages of outbreak
Sambaraju et al. (2012)	6000	Western Canada	Finds that the most important variable in a statistical model is infestation in the previous year within the 12 km x 12 km cells
Howe et al. (2021)	5000	British Columbia	Finds that a distance weighted beetle pressure metric from the previous year within 5 km provided the best explanatory power for a statistical model fit to infestation data
Carroll et al. (2017)	2000	Alberta	Finds that more than 75% of new infestations occur within 2 km of "parent" polygons containing the infestations from the previous year using heli-GPS data
Simard et al. (2012)	2000	Wyoming	Finds beetle pressure, up to 2 km, is the most important predictor for subsequent outbreaks for mountain pine beetle, spruce beetle, and Douglas-fir beetle
Robertson et al. (2009)	1000	Canadian Rocky Mountains	Finds that most movement distances centre around 1 km using spatial–temporal analysis of moving polygons (STAMP)
Strohm et al. (2013)	364	Sawtooth National Recreation Area, Idaho	Finds MPB attacks should be spaced by 364 m by studying pattern formation in a model of chemical signalling including diffusion and chemotaxis and parameterized with data from Biesinger et al. (2000)
Powell and Bentz (2014)	5 – 90	Sawtooth National Recreation Area, Idaho	Finds that density-dependent motility varies from an unimpeded motility of 3.79 km^2 per day to 18.5 m^2 per day in a fully stocked stand by fitting a phenology and dispersal model to aerial detection data
Robertson et al. (2007)	30 – 50	British Columbia	Finds that the most common distances between newly and previously attacked trees are 30 m and 50 m given a search radius of 100 m from previously attacked trees
Safranyik et al. (1992)	30	British Columbia	Finds that 86% and 93% of total captured beetles were found within 30 m of release site in a mark-recapture experiment in two years
Heavilin and Powell (2008)	10 – 15	Sawtooth National Recreation Area, Idaho	Finds a mean and median dispersal distance of between 10 m to 15 m by fitting Gaussian and exponential dispersal kernels to aerial detection data
Goodsman et al. (2016)	10	British Columbia and Alberta	Finds a median dispersal distance of 10 m by parameterizing a 2D dispersal kernel with mark-recapture data from Safranyik et al. (1992)

Table S4: Additional information on the stated values in Table 1 for related bark beetles. This includes information about the study location as well as more details on where we obtained the characteristic scale of infestations.

Study	Scale (m)	Study location	Bark beetle species	Source of scale
Withrow et al. (2013)	1000 – 2500	Colorado and Wyoming	Douglas-fir beetle	Finds average standard dispersal distances — distances at which 68% of infestations dispersed in a given year — between 1000 m and 2500 m by quantifying the distance between infestations and modeling these distances using a cumulative Gaussian function
Turchin and Thoeny (1993)	690	Louisiana	Southern pine beetle	Finds a median dispersal distance of 690 m for released beetles by fitting a dispersal model with mark-recapture data
Werner and Holsten (1997)	90 – 300	Alaska	Spruce beetle	Finds that most recaptured beetles from standing trees dispersed between 90 m and 300 m in mark-recapture experiments
Zumr (1992)	200	Southern Bohemia	European spruce beetle	Finds that most beetles (about 70%) were captured within 200 m of release in mark-recapture experiments
Dodds and Ross (2002)	200	Idaho	Douglas-fir beetle	Finds that most beetles (over 90%) were captured within 200 m of release in mark-recapture experiments
Kautz et al. (2011)	100	Bavaria	European spruce beetle	Finds that 65% of new infestations occurred within 100 m of the previous year's infestations
Zolubas and Byers (1995)	10	Western Lithuania	European spruce beetle	Finds that most beetles (about 67%) were captured within 10 m of release in mark-recapture experiments for the second flight

Section S4 Additional figures & tables

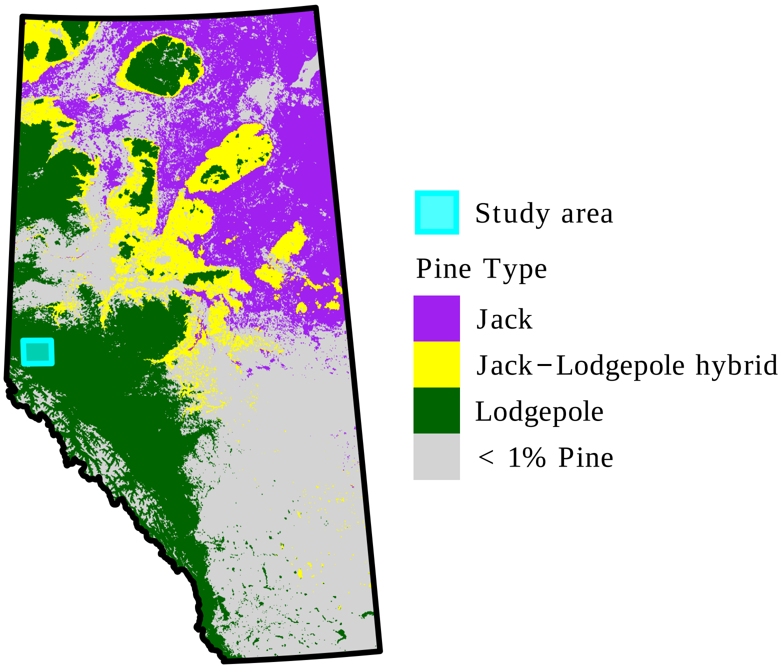

Figure S3: Location of our study area in Alberta. Our study area (cyan) is an approximately 2,500 km^2 block of high-biomass lodgepole pine, and was surveyed annually from 2006–2021. Pine species data comes from Cullingham et al. (2012). The grey pixels are areas where pines constitute less than 1% of total live aboveground biomass; data from Beaudoin et al. (2014).

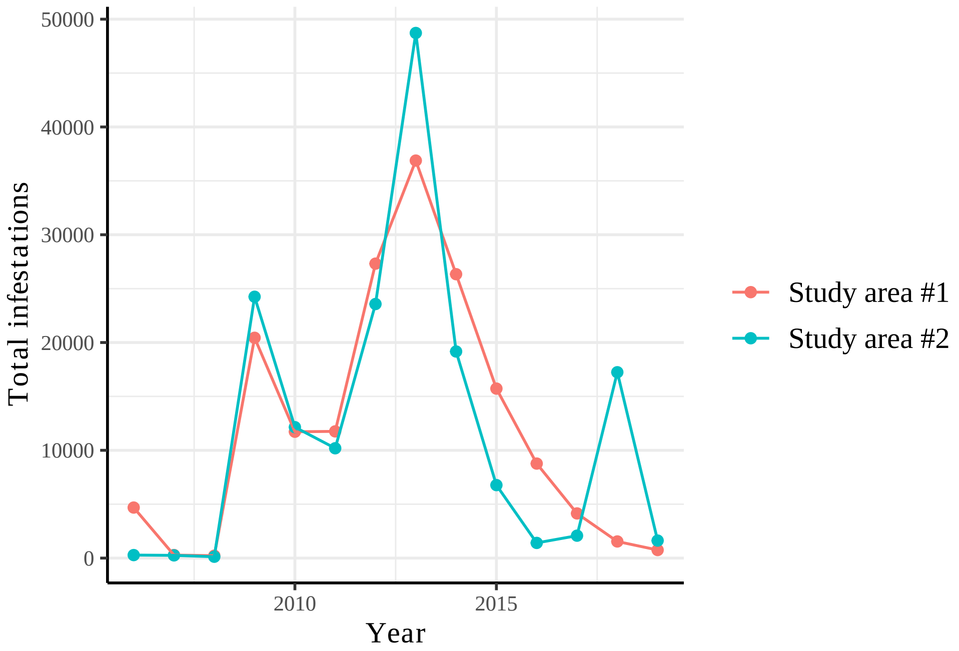

Figure S4: Time series of total infestations in both study areas. A large number of beetles were present from 2009–2019.

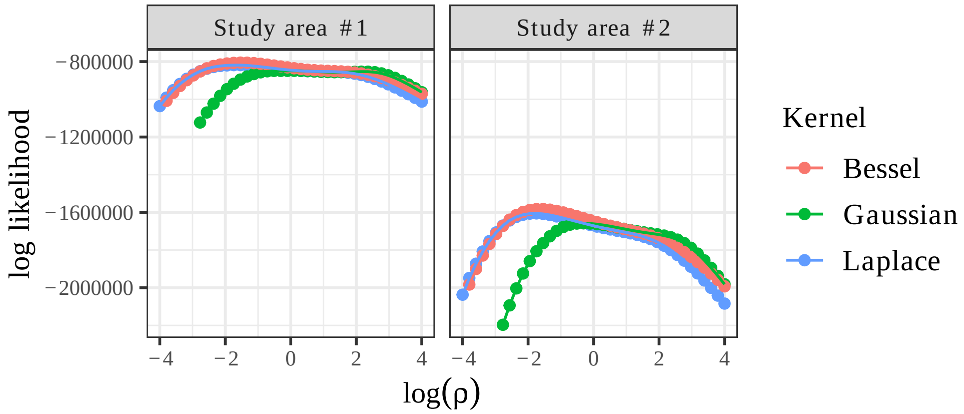

Figure S5: Likelihood profiles for the three one-parameter redistribution models. The gradient of the log likelihood is relatively flat for large values of the scale parameter ρ, which can cause the failure of gradient-based optimization methods. Instead, we used the grid search method to find the Maximum Likelihood Estimates for these models.

Table S5: Parameter values for the redistribution models, fit to data from study area #1. The parameters ρ, ρ_1, and ρ_2 have units of kilometers; the remaining parameters are dimensionless.

Kernel name	Tail type	$D(r) \propto$	Max likelihood estimate
Pareto	Fat-tail	$(r+\rho)^{-(1+\nu)}$	$\rho = 1.35 \cdot 10^{-2},\ \nu = 1.57$
Student's t	Fat-tail	$\left(1+\frac{1}{\nu}\left(\frac{r}{\rho}\right)^2\right)^{-\frac{\nu+1}{2}}$	$\rho = 1.18 \cdot 10^{-2},\ \nu = 1.45$
Bessel mixture	Mixture of Thin	$\theta\, K_0(\frac{r}{\rho_1}) + (1-\theta)K_0(\frac{r}{\rho_2})$	$\rho_1 = 3.20 \cdot 10^{-2},\ \rho_2 = 4.62,\ \theta = 6.56 \cdot 10^{-1}$
Laplace mixture	Mixture of Thin	$\theta \exp\left(-\frac{r}{\rho_1}\right) + (1-\theta)\exp\left(-\frac{r}{\rho_2}\right)$	$\rho_1 = 2.12 \cdot 10^{-2},\ \rho_2 = 3.91,\ \theta = 6.18 \cdot 10^{-1}$
Gaussian mixture	Mixture of Thin	$\theta \exp\left(-\left(\frac{r}{\rho_1}\right)^2\right) + (1-\theta)\exp\left(-\left(\frac{r}{\rho_2}\right)^2\right)$	$\rho_1 = 2.71 \cdot 10^{-2},\ \rho_2 = 1.03 \cdot 10^{1},\ \theta = 4.91 \cdot 10^{-1}$
WMY	Thin tail	$\left(\frac{r}{\rho}\right)^{\kappa} K_{\kappa}(\frac{r}{\rho})$	$\rho = 2.26 \cdot 10^{-1},\ \kappa = 3.95 \cdot 10^{-9}$
Bessel	Thin tail	$K_0(\frac{r}{\rho})$	$\rho = 2.15 \cdot 10^{-1}$
Laplace	Thin tail	$\exp\left(-\frac{r}{\rho}\right)$	$\rho = 2.15 \cdot 10^{-1}$
Gaussian	Thin tail	$\exp\left(-\left(\frac{r}{\rho}\right)^2\right)$	$\rho = 1.11$

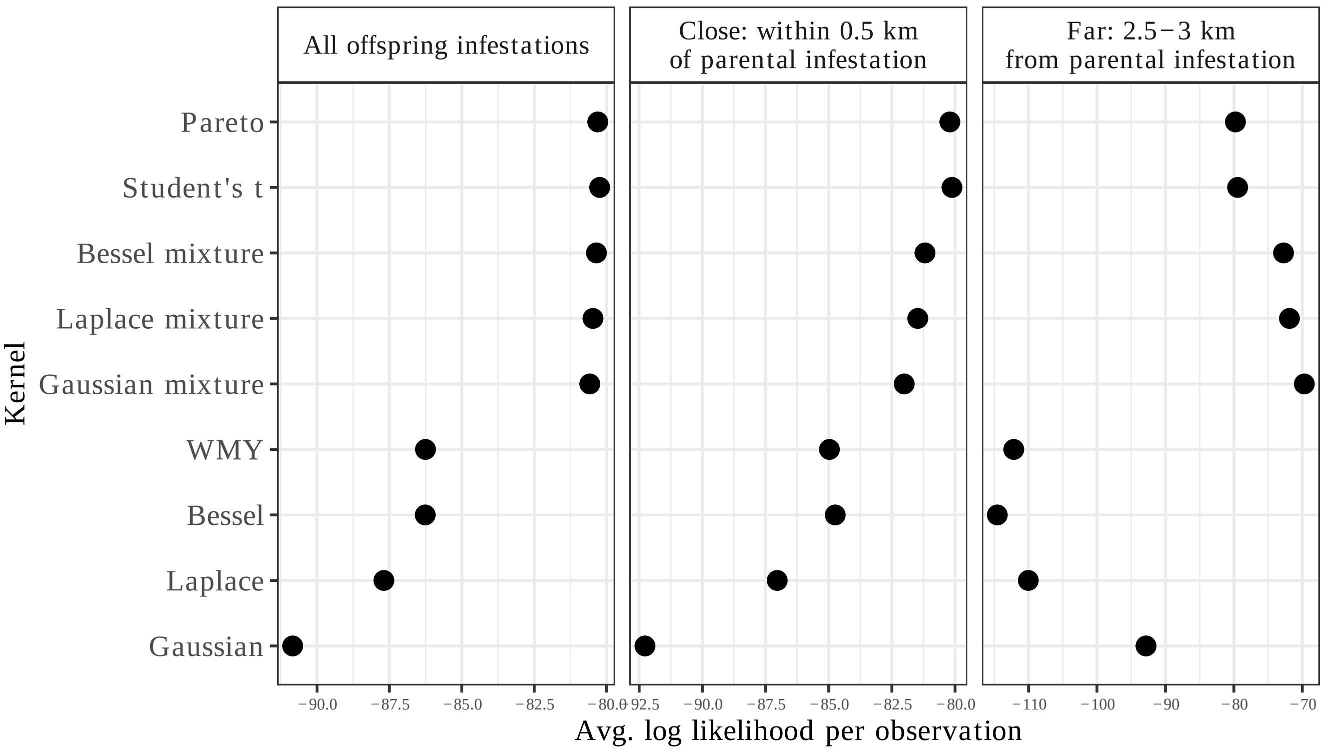

Figure S6: Model fit for observations that are various distances from the nearest parental infestations. Observations that were within 0.5 kilometers of the nearest parental infestation (representing short-to-medium distance dispersal) were best modeled by the fat-tailed distributions, the Pareto and Student's t-distributions. Observations that were within 2.5–3 km from the nearest parental observation were best modeled by the mixture distributions.

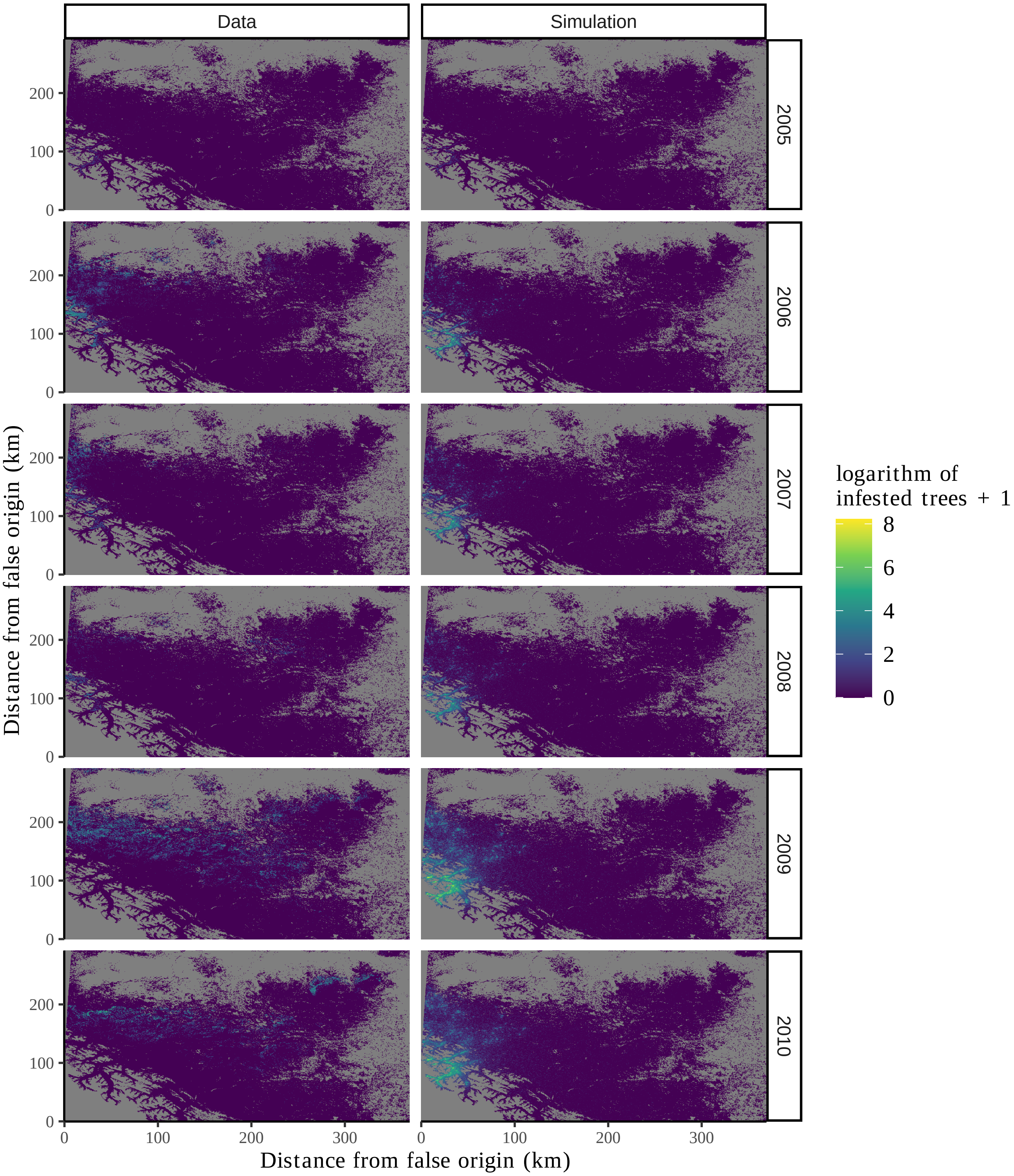

Figure S7: Comparison of the spatial distribution of infestations for both real and simulated data. Our simulations do not account for resource depletion, which leads to an unrealistic concentration of future infestations around the initial infestations. Our simulations do not account for beetle aggregation or clumpy dispersal, which leads to an unrealistically high spatial dispersion of infestations.

References

Aukema, B. H., Carroll, A. L., Zheng, Y., Zhu, J., Raffa, K. F., Dan Moore, R., Stahl, K., and Taylor, S. W. (2008). Movement of outbreak populations of mountain pine beetle: Influences of spatiotemporal patterns and climate. *Ecography*, 31(3):348–358.

Beaudoin, A., Bernier, P., Guindon, L., Villemaire, P., Guo, X., Stinson, G., Bergeron, T., Magnussen, S., and Hall, R. (2014). Mapping attributes of canada's forests at moderate resolution through k nn and modis imagery. *Canadian Journal of Forest Research*, 44(5):521–532.

Biesinger, Z., Powell, J., Bentz, B., and Logan, J. A. (2000). Direct and indirect parametrization of a localized model for the mountain pine beetle — lodgepole pine system. *Ecological Modelling*, 129(2):273–296.

Carroll, A., Seely, B., Welham, C., and Nelson, H. (2017). Assessing the effectiveness of Alberta's forest management program against the mountain pine beetle: Final report for fRI research project 246.18 parts 1 and 2. Technical report, fRI Research.

Cullingham, C. I., James, P. M., Cooke, J. E., and Coltman, D. W. (2012). Characterizing the physical and genetic structure of the lodgepole pine× jack pine hybrid zone: Mosaic structure and differential introgression. *Evolutionary applications*, 5(8):879–891.

Dodds, K. J. and Ross, D. W. (2002). Sampling range and range of attraction of *Dendroctonus pseudotsugae* pheromone-baited traps. *The Canadian Entomologist*, 134(3):343–355.

Goodsman, D. W., Koch, D., Whitehouse, C., Evenden, M. L., Cooke, B. J., and Lewis, M. A. (2016). Aggregation and a strong Allee effect in a cooperative outbreak insect. *Ecological Applications*, 26(8):2623–2636.

Heavilin, J. and Powell, J. A. (2008). A novel method of fitting spatio-temporal models to data, with applications to the dynamics of mountain pine beetles. *Natural Resource Modeling*, 21(4):489–524.

Howe, M., Carroll, A., Gratton, C., and Raffa, K. F. (2021). Climate-induced outbreaks in high-elevation pines are driven primarily by immigration of bark beetles from historical hosts. *Global Change Biology*, 27(22):5786–5805.

Kautz, M., Dworschak, K., Gruppe, A., and Schopf, R. (2011). Quantifying spatio-temporal dispersion of bark beetle infestations in epidemic and non-epidemic conditions. *Forest Ecology and Management*, 262(4):598–608.

Koch, D., Lewis, M. A., and Lele, S. (2021). The signature of endemic populations in the spread of mountain pine beetle outbreaks. *Bulletin of Mathematical Biology*, 83(6):65.

Powell, J. A. and Bentz, B. J. (2014). Phenology and density-dependent dispersal predict patterns of mountain pine beetle (*Dendroctonus ponderosae*) impact. *Ecological Modelling*, 273:173–185.

Preisler, H. K., Hicke, J. A., Ager, A. A., and Hayes, J. L. (2012). Climate and weather influences on spatial temporal patterns of mountain pine beetle populations in Washington and Oregon. *Ecology*, 93(11):2421–2434.

Robertson, C., Nelson, T. A., and Boots, B. (2007). Mountain pine beetle dispersal: The spatial-temporal interaction of infestations. *Forest Science*, 53(3):395–405.

Robertson, C., Nelson, T. A., Jelinski, D. E., Wulder, M. A., and Boots, B. (2009). Spatial–temporal analysis of species range expansion: The case of the mountain pine beetle, *Dendroctonus ponderosae*. *Journal of Biogeography*, 36(8):1446–1458.

Safranyik, L., Linton, D. A., Silversides, R., and McMullen, L. H. (1992). Dispersal of released mountain pine beetles under the canopy of a mature lodgepole pine stand. *Journal of Applied Entomology*, 113(1-5):441–450.

Sambaraju, K. R., Carroll, A. L., Zhu, J., Stahl, K., Moore, R. D., and Aukema, B. H. (2012). Climate change could alter the distribution of mountain pine beetle outbreaks in western Canada. *Ecography*, 35(3):211–223.

Simard, M., Powell, E. N., Raffa, K. F., and Turner, M. G. (2012). What explains landscape patterns of tree mortality caused by bark beetle outbreaks in Greater Yellowstone? *Global Ecology and Biogeography*, 21(5):556–567.

Strohm, S., Tyson, R. C., and Powell, J. A. (2013). Pattern formation in a model for mountain pine beetle dispersal: Linking model predictions to data. *Bulletin of Mathematical Biology*, 75(10):1778–1797.

Turchin, P. and Thoeny, W. T. (1993). Quantifying dispersal of southern pine beetles with mark-recapture experiments and a diffusion model. *Ecological Applications*, 3(1):187–198.

Werner, R. A. and Holsten, E. H. (1997). Dispersal of the spruce beetle, *Dendroctonus rufipennis*, and the engraver beetle, *Ips perturbatus*, in Alaska. Research Paper PNW-RP-501, U.S. Department of Agriculture, Forest Service, Pacific Northwest Research Station, Portland, OR.

Withrow, J. R., Lundquist, J. E., and Negrón, J. F. (2013). Spatial dispersal of Douglas-fir beetle populations in Colorado and Wyoming. *ISRN Forestry*, 2013:1–10.

Zolubas, P. and Byers, J. A. (1995). Recapture of dispersing bark beetle *Ips typographus* L. (*Col.*, *Scolytidae*) in pheromone-baited traps: Regression models. *Journal of Applied Entomology*, 119(1-5):285–289.

Zumr, V. (1992). Dispersal of the spruce bark beetle *Ips typographus* (L.) (*Col.*, *Scolytidae*) in spruce woods. *Journal of Applied Entomology*, 114(1-5):348–352.
